# Reading Between the ABCs: Intrinsic Disorder and Evolutionary Dynamics of Non-Canonical Regions in ABC Transporters

**DOI:** 10.3390/ijms27114699

**Published:** 2026-05-23

**Authors:** Ichda Arini Dinana, Yukihiko Kubota, Masahiro Ito

**Affiliations:** 1 Advanced Life Sciences Program, Graduate School of Life Sciences, Ritsumeikan University, 1-1-1 Nojihigashi, Kusatsu 525-8577, Shiga, Japan; ichdaarini@gmail.com; 2 Department of Bioinformatics, College of Life Sciences, Ritsumeikan University, 1-1-1 Nojihigashi, Kusatsu 525-8577, Shiga, Japan; yukubota@fc.ritsumei.ac.jp

**Keywords:** ABC transporter, intrinsic disorder, post-translational modification, non-canonical regions, evolutionary dynamics, linker regions, phylogenetic signal, site-specific selection, architectural class

## Abstract

ATP-binding cassette (ABC) transporters are one of the largest superfamilies of membrane proteins, but little is known about the structural and evolutionary features of their non-domain regions. To clarify the diversity of these non-canonical regions across evolutionary lineages, we performed an analysis of intrinsically disordered regions, site-specific selection and predicted post-translational modification (PTM) sites among five architectural classes involving 1581 prokaryotic and eukaryotic sequences. Linker and flanking regions were more disordered than transmembrane and nucleotide-binding domains in all architectures. Disorder fraction was significantly different between region types after phylogenetic correction (Pagel’s λ ≈ 0.97). Predicted PTM sites are enriched in disordered non-domain segments, with N-linked glycosylation and phosphoserine showing the strongest positive enrichment. A total of 140 sites satisfied a tiered conservation criterion (MusiteDeep score ≥ 0.5; cross-species conservancy ≥ 30%), including 40 high-confidence or moderate-confidence sites (conservancy ≥ 50%) as well as novel phosphotyrosine candidates in half transporters and NBD-only proteins. Site-specific selection analyses showed pervasive purifying selection across domain cores and architecture-dependent enrichment of episodic positive selection in non-domain regions, with significant non-domain enrichment in full reverse and half forward transporters (Fisher’s exact, BH-adjusted *p* < 0.05). In summary, these findings establish that non-canonical regions of ABC transporters are evolutionarily dynamic and contain conserved predicted modification sites, supporting the idea that these regions are evolutionary dynamic segments that deserve experimental characterization as candidate regulatory interfaces.

## 1. Introduction

ATP-binding cassette (ABC) transporters constitute one of the largest and most phylogenetically widespread superfamilies of membrane proteins, with members found in organisms ranging from bacteria to humans [1,2]. These proteins function as primary active transporters, using ATP hydrolysis to translocate a variety of substrates, including lipids, ions, peptides, and xenobiotics, across the cellular membrane [3,4]. In humans, 48 members are classified into seven subfamilies (ABCA–ABCG) based on sequence similarity and domain architecture [1,5]. The basic structural unit of ABC transporters consists of transmembrane domains (TMDs) and nucleotide-binding domains (NBDs), which can be arranged as full transporters within a single polypeptide (TMD–NBD–TMD–NBD), as half transporters that must dimerize (TMD–NBD or NBD–TMD), or as soluble proteins containing only NBDs and lacking transmembrane segments [2,3,6,7]. The domain topology is typically conserved, with most subfamilies presenting a forward TMD–NBD configuration, except for ABCG members, which have a reversed NBD–TMD configuration [8,9]. NBDs are the most conserved parts of this overall architecture, with Walker A, Walker B, and LSGGQ signature motifs, while TMDs are more variable and largely define substrate specificity [2,10].

In addition to these canonical domains, ABC transporters harbor inter-domain linkers and N- and C-terminal flanking regions whose structural and evolutionary characteristics have been relatively less studied. Ford et al. [11] reviewed the disordered linker regions in eukaryotic ABC transporters and proposed that these segments could be regulated by post-translational modifications (PTMs), particularly phosphorylation. The best characterized example is the regulatory R-domain of CFTR (ABCC7), where phosphorylation of multiple serine residues in a disordered ~200-residue linker between NBD1 and TMD2 governs channel gating through disorder-dependent conformational switching [12,13]. Regulation by phosphorylation of non-domain regions in the cytosol has been reported for other ABC transporters. In ABCC1, PTM sites located in the intrinsically disordered L1 linker modulate protein–protein interactions and transport activity [14,15]. In ABCA1, CK2-mediated phosphorylation of residues in the R1 inter-domain linker decreases cholesterol efflux and apoA-I binding [16], whereas JAK2-mediated tyrosine phosphorylation increases cholesterol efflux in response to apoA-I stimulation [17]. The conservation of comparable non-domain PTM landscapes across all ABC transporter architectures and their shaping by evolutionary constraints have not been studied at the superfamily level so far.

Studies of intrinsically disordered regions (IDRs) in other multidomain protein families provide the wider context for this question. Holehouse & Kragelund [18] demonstrated that linker IDRs between folded domains influence local domain concentrations and control inter-domain interactions in a length and sequence-dependent manner, with PTMs providing a mechanism for dynamic regulation. IDRs are also enriched in PTM sites relative to structured domains across multiple species, suggesting that such enrichment is a general feature of disordered protein segments rather than a family-specific feature [19,20]. Evolutionarily, IDRs typically evolve more rapidly than ordered domains due to their less constrained structure. Nevertheless, their biophysical properties, such as disorder propensity and compositional bias, can be phylogenetically conserved despite primary-sequence divergence [21,22]. Whether non-domain regions of ABC transporters have the same patterns remains unclear. The differences in structural properties among the five architectural classes have not been characterized. These questions have not been systematically investigated.

Here we describe the structural and evolutionary characteristics of non-canonical regions across the ABC transporter superfamily by analyzing intrinsic disorder, site-specific selection, and predicted PTM sites across five architectural classes comprising 1581 prokaryotic and eukaryotic sequences. We find that non-domain regions are consistently more disordered than domain cores for all architectures, are enriched in predicted PTM sites that are partially conserved across species, and experience distinctive evolutionary pressures. Episodic positive selection, specifically, is focused on the inter-domain boundaries, while NBD cores are under widespread purifying selection. These results provide a systematic view of the non-canonical sequence space of ABC transporters and highlight potential regulatory regions for future experimental investigation.

## 2. Results

### 2.1. Domain Architecture and Transmembrane Profiles Across ABC Subfamilies

**Figure 1:**
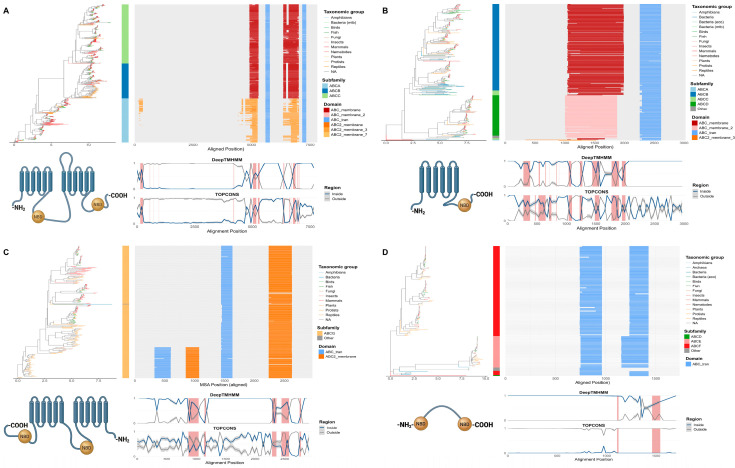
Domain architecture and transmembrane topology across ABC transporter architectural classes. Sequences are ordered by phylogenetic topology; branch colours indicate taxonomic group; vertical strips indicate subfamily membership. Main panel: HMMER-annotated domain composition per sequence (ABC_membrane variants, orange/red; ABC_tran, blue). Bottom left: topology schematic showing TMD (blue), NBD (yellow), and N-/C-terminal orientations. Bottom right: consensus transmembrane helix occupancy profile (DeepTMHMM and TOPCONS); red shading ≥ 50% TM occupancy. (**A**) Full forward. (**B**) Half forward. (**C**) Full and half reverse. (**D**) NBD-only.

To characterize the structural organization of ABC transporters across evolutionary lineages, 1581 prokaryotic and eukaryotic sequences were classified into five architectural classes using HMM-based domain annotation and transmembrane topology prediction (Figure 1; Supplementary Table S2).

Full forward transporters (TMD–NBD–TMD–NBD; *n* = 751) were classified into the ABCA, ABCB and ABCC subfamilies (Figure 1A). TMD2 showed annotation heterogeneity across subfamily members, with ABC2_membrane, ABC2_membrane_3, and ABC2_membrane_7 variants detected, reflecting the structural diversity in C-terminal TMD configurations in ABCA and ABCC members. Some of the ABCC sequences had another N-terminal TMD0 domain corresponding to the regulatory transmembrane domain found in long MRP-type transporters, and exclusive to the mammalian and vertebrate lineages. Both DeepTMHMM and TOPCONS predicted two concordant clusters of transmembrane helices, TMD1 in the N-terminal third of the alignment and TMD2 in the C-terminal third, respectively. Expected for cytoplasmic location, low transmembrane signal was detected for NBD regions and inter-domain linkers. Profiles of the inside/outside topology further supported an extracellular N-terminus and NBDs in the cytoplasm.

Half forward transporters (TMD–NBD; *n* = 372) of ABCB and ABCD members are shown as single polypeptides that require homo- or heterodimerization (Figure 1B). Both predictors identified a single cluster of transmembrane helices in the N-terminal half of the alignment and no membrane association in the C-terminal NBD region, consistent with the canonical six-helix TMD bundle. Phylogenetic mixing of prokaryotic and eukaryotic sequences suggests an early evolutionary origin of the half-transporter configuration.

Full and half reverse transporters (NBD–TMD; *n* = 69 full, *n* = 228 half) were confined to the ABCG subfamily (Figure 1C). In these proteins ABC_tran domains are followed by ABC2_membrane domains. DeepTMHMM and TOPCONS profiles confirmed transmembrane helix predictions shifted towards the C-terminus, with no membrane association in the N-terminal NBD region. Inside/outside topology profiles demonstrated a cytoplasmic N-terminus—the opposite of forward transporter topology—consistent with the known architecture of ABCG2 (BCRP) and the obligatory heterodimer ABCG5/G8.

NBD Only proteins group (*n* = 154) representing ABCE and ABCF lacked any membrane-associated domain annotations (Figure 1D). Both predictors failed to identify any transmembrane regions at any position, confirming their soluble cytoplasmic nature. Both predictors showed flat probability profiles near zero across the entire alignment.

### 2.2. Disorder Propensity Across ABC Transporter Classes

**Figure 2:**
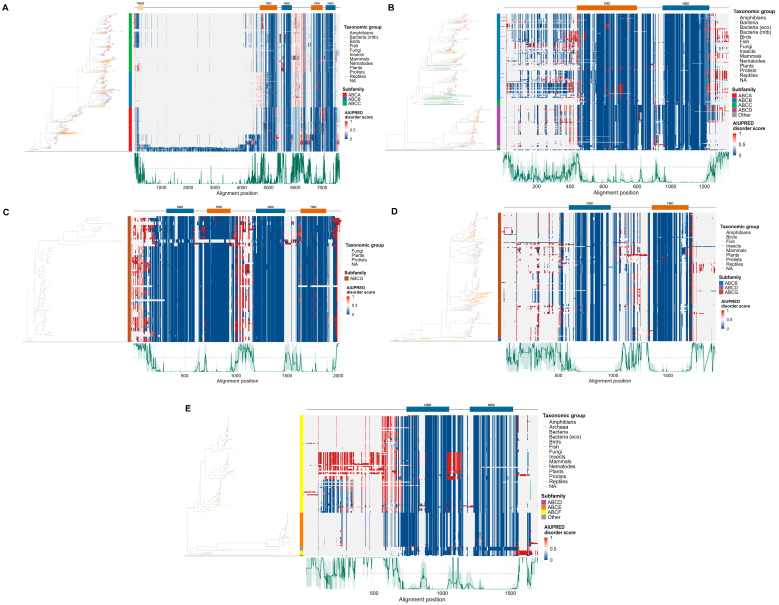
Intrinsic disorder heatmap across ABC transporter architectural classes. For each panel, sequences are ordered by phylogenetic topology. Main panel: per-position AIUPred disorder score mapped onto multiple sequence alignment coordinates; blue = ordered (score 0); red = disordered (score 1); grey = alignment gap; color scale shown at right with 0.5 threshold indicated. Domain annotation track (top): TMD regions (orange) and NBD regions (blue) with boundaries derived from HMMER coordinates. Mean disorder track (bottom): mean AIUPred disorder ± 1 SD per alignment position (dark green line; shaded band); dashed horizontal line = 0.5 disorder threshold. (**A**) Full forward transporters. (**B**) Half forward transporters. (**C**) Full reverse transporters. (**D**) Half reverse transporters. (**E**) NBD-only soluble proteins.

**Figure 3:**
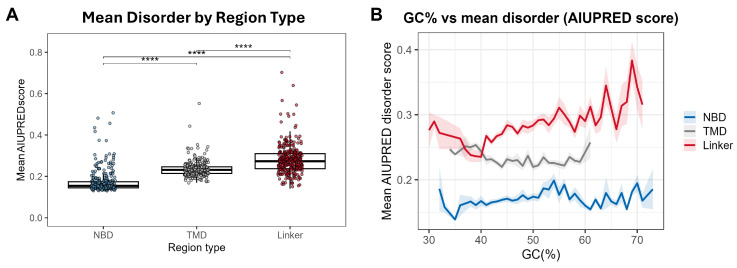
Intrinsic disorder hierarchy across region types and its relationship with genomic GC content. (**A**) Mean AIUPred disorder score per gene-level region grouped by region type; individual data points shown; box plots show median and interquartile range; significance brackets indicate BH-adjusted pairwise Wilcoxon comparisons (**** *p* < 0.0001). (**B**) Binned means AIUPred disorder score as a function of GC content (30–75%) stratified by region type (NBD, blue; TMD, grey; linker, red); shaded bands = 95% CI; This positive GC–disorder relationship in linker regions was preserved under binary thresholding of disorder scores (Supplementary Figure S3), confirming that the trend is not an artefact of the continuous scoring metric.

**Figure 4:**
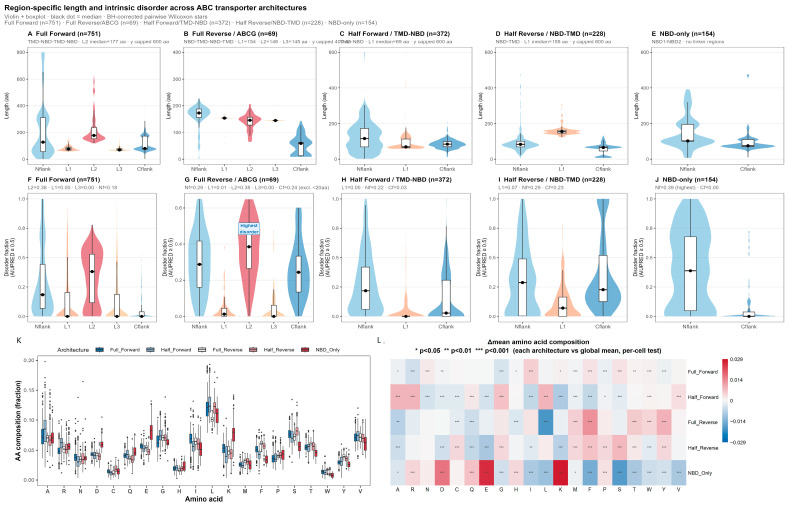
Region-specific length and intrinsic disorder of non-canonical regions across ABC transporter architectural classes. Violin plots with embedded box plots; black dot = median; significance brackets indicate BH-corrected pairwise Wilcoxon comparisons. Pink violins = inter-domain linker regions; blue violins = N- and C-terminal flanking regions. Top row (**A**–**E**): region length (amino acids; *y*-axis capped as indicated). Bottom row (**F**–**J**): disorder fraction, defined as the proportion of residues with AIUPred score ≥ 0.5. (**A**,**F**) Full forward transporters (TMD–NBD–TMD–NBD; *n* = 751): L2 is the dominant disordered linker (median length 177 aa, median disorder fraction 0.38); L1, L3, and Cflank are largely ordered (median disorder ≈ 0.00); Nflank shows moderate disorder (median 0.18). (**B**,**G**) Full reverse transporters (NBD–TMD–NBD–TMD; ABCG subfamily; *n* = 69): L1, L2, and L3 are of comparable length (medians 154, 146, and 145 aa, respectively); L2 carries the highest disorder (median 0.38), while L1 and L3 are largely ordered (median ≤ 0.01); Nflank disorder median 0.29. (**C**,**H**) Half forward transporters (TMD–NBD; *n* = 372): single inter-domain linker median length 69 aa, median disorder fraction 0.00; Nflank median disorder 0.22; Cflank median 0.03. (**D**,**I**) Half reverse transporters (NBD–TMD; *n* = 228): single inter-domain linker median length 155 aa, median disorder fraction 0.07; Nflank median disorder 0.29; Cflank median 0.23. (**E**,**J**) NBD-only soluble proteins (*n* = 154): no inter-domain linker regions present; Nflank carries the highest median disorder fraction among all regions and architectures examined (median 0.39); Cflank is largely ordered (median ≈ 0.00). (**K**) Per-residue amino acid composition (fraction) stratified by architectural class. (**L**) Δmean amino acid composition heatmap showing deviation from the global mean per residue.

To characterize structural features of ABC transporter sequences beyond annotated domain cores, per-residue intrinsic disorder was predicted across all five architectural classes using AIUPred, and secondary structure propensity was independently predicted using NetSurfP-3.0. Disorder scores were visualized as phylogenetically ordered heatmaps with corresponding mean disorder profiles (Figure 2), and region-specific disorder fractions were quantified at the gene level (Figure 3 and Figure 4). Secondary structure profiles confirmed that annotated NBD and TMD cores consistently show high helical and sheet propensities, whereas inter-domain linker and flanking regions are coil-dominant across all architectural classes, corroborating the AIUPred predictions (see Supplementary Figure S1).

Full forward transporters showed elevated disorder scores (Figure 2A) at three positional intervals: the N-terminal pre-TMD0 segment in long ABCC members, the L2 linker between NBD1 and TMD2, and C-terminal extensions beyond NBD2. Within ABCC sequences, the TMD0 helix bundle was a locally ordered region, and the L0 linker and NBD1-TMD2 regulatory region were highly disordered across most sequences. This is consistent with crystallographic and cryo-EM data that show these segments are poorly resolved in structural studies [12,23] and reflect conformational heterogeneity rather than independently confirming intrinsic disorder. However, several lines of biochemical and structural evidence support real disorder in these linker regions, including phosphorylation-dependent conformational changes mediated by IDRs in several ABCC members [24] and NMR and HDX studies confirming that the CFTR R-domain is disordered in isolation, gaining transient helical structure only upon phosphorylation [13].

In ABCA and ABCB sequences, the disorder was more limited to inter-domain linker sites and terminal flanking regions. Quantification showed that L2 had the highest median disorder fraction of all linker segments in full forward transporters (median 0.38), while L1, L3 and Cflank were largely ordered (median approximately 0.00), and Nflank had moderate disorder (median 0.19; Figure 4A,F). In the complete forward class, the L2 region showed significant subfamily differences. ABCC members had the highest L2 disorder (median 0.526) with a median length of 158 aa, ABCB had intermediate disorder (median 0.395, length 158 aa), while ABCA members had the longest L2 in the dataset (median 303 aa) but the lowest disorder fraction (median 0.083), suggesting that in the ABCA subfamily, this segment may play a structural rather than regulatory role. This structured character might be due to the ordered fold of the large extracellular domains (~600 aa each) characteristic of ABCA members, which are revealed in available cryo-EM structures [25]. The L2 disorder fraction differed significantly among the three major subfamilies of the full forward class (Kruskal–Wallis H = 364.0, *p* = 1.01 × 10^−79^; Supplementary Figure S2). All pairwise comparisons were significant (BH-adjusted *p* < 0.0001): ABCC had the highest disorder (median 0.526; Cliff’s δ = −0.903 vs. ABCA, large effect), ABCB was intermediate (median 0.395; δ = −0.416 vs. ABCC, medium), and ABCA was lowest (median 0.083; δ = −0.625 vs. ABCB, large).

Half-forward transporters exhibited a more compact disorder pattern, with more disorder in the N-terminal and C-terminal flanking regions (Nflank median 0.29; Cflank median 0.23) and in the NBD–TMD linker (Figure 2B and Figure 4C,H). The median disorder fraction for the single inter-domain linker was 0.07, and the median length was 155 aa. In ABCB half transporters, this region was more disordered, in line with the intracellular loop architecture of P-glycoprotein-related proteins [26]. ABCD half transporters had a similar profile.

Full reverse transporters showed a distributed disorder pattern over several non-domain segments, compared to a single dominant disordered linker in full forward transporters (Figure 2C and Figure 4B,G). Disorder was increased in the N-terminal flanking region before NBD1 (Nflank median disorder fraction 0.288), in the L2 linker between TMD1 and NBD2 (median 0.385, length 146 aa) and in the C-terminal flanking region after TMD2 (Cflank median 0.355; note that 36% of full reverse sequences have Cflank lengths below 20 aa and the disorder fraction for sequences with Cflank ≥ 20 aa is 0.244). The intervening linkers L1 and L3, connecting NBD1 to TMD1 and NBD2 to TMD2, respectively, were predominantly ordered (median disorder fraction ≤ 0.013; Figure 4G). The multi-segment disorder distribution reflects the inverted NBD–TMD–NBD–TMD topology of ABCG members where the absence of a single inter-domain gap is long enough to serve as a dedicated regulatory linker. A formal comparison between the full reverse and half reverse transporters, both consisting of members of the ABCG subfamily, confirmed the linker disorder fraction was significantly higher in the full reverse class (median 0.385 vs. 0.071; Mann–Whitney U, *p* = 9.51 × 10^−23^; Cliff’s δ = 0.775, large effect; Supplementary Table S3). Disorder was also elevated in cflank disorder of full reverse transporters (median 0.355 vs. 0.227; *p* = 0.008; δ = 0.209, small) while overall disorder fraction (*p* = 0.198) and Nflank disorder (*p* = 0.344) did not differ suggesting that the architectural difference is linker-specific and not affecting global protein disorder. Half reverse transporters exhibited a simpler profile with moderate disorder in the N-terminal flank (median 0.218) and near-zero disorder in the single TMD–NBD linker (median 0.000; Figure 2D and Figure 4D,I).

By definition, NBD-only proteins did not have transmembrane-flanking disorder, as these classes do not carry TM segments. Disorder was localized to the inter-NBD linker and terminal extensions, with the Nflank showing the highest median disorder fraction recorded across all regions and architectures (median 0.388) while the Cflank remained largely ordered (median 0.000; Figure 2E and Figure 4E,J).

Across all architectural classes, linker and flanking regions were significantly more disordered than TMD and NBD cores (BH-adjusted pairwise Wilcoxon, all comparisons *p* < 0.0001; Figure 3A). PGLS models incorporating branch-length-scaled covariance yielded Pagel’s λ of 0.955–0.972 across model specifications (Table 1), confirming strong phylogenetic signal in linker disorder and indicating that the observed hierarchy is phylogenetically conserved rather than an artefact of shared ancestry alone.

The best-fitting PGLS model incorporated amino acid composition principal components with GC2% as predictors (M2: AIC = −6554.1, adj. R^2^ = 0.426; Table 1), with amino acid composition alone accounting for most of the explained variance (M3: adj. R^2^ = 0.390; AIC = −6458.5; ΔAIC M2 vs. M3 = −95.6). Among the four GC predictors tested individually, GC2% was the strongest single predictor of linker disorder (M1: adj. R^2^ = 0.164), followed by GC1% (0.081), total GC% (0.072), and GC3% (0.036), This pattern indicates that the GC–disorder association is driven primarily by non-synonymous codon positions via amino acid composition.

Region-specific quantification across architectural classes further resolved these patterns (Figure 4A–J; Supplementary Table S4). In half forward transporters (TMD–NBD; *n* = 372) the single inter-domain linker had a median disorder fraction of 0.07, while in half reverse transporters (NBD–TMD; *n* = 228) the linker was mostly ordered (median 0.00; Supplementary Table S4). No significant difference in Nflank disorder was observed between any pairwise comparison of architectural classes (all BH-adjusted *p* > 0.05; Cliff’s δ −0.148 to +0.128; Supplementary Table S4). Statistical power was sufficient to detect medium-to-large effect sizes with sample sizes per class (*n* = 69–751), and the consistently small effect sizes suggest that elevated Nflank disorder is a bona fide architecture-independent property, perhaps reflecting a conserved role in N-terminal flexibility or co-translational membrane targeting across all ABC transporter classes.

The amino acid composition of linker regions was highly variable among architectural classes (Figure 4, bottom panels). Full reverse transporters were enriched in lysine and depleted in isoleucine compared with the global mean (permutation FDR *p* < 0.001 for both), and NBD-only proteins were most divergent in composition overall, with strong enrichment of glutamate and lysine. Glutamate and lysine are both known disorder-promoting residues [27,28,29], consistent with the observed differences in disorder architecture. Full statistical annotation per amino acid and architecture are presented in Supplementary Figure S4.

**Figure 5:**
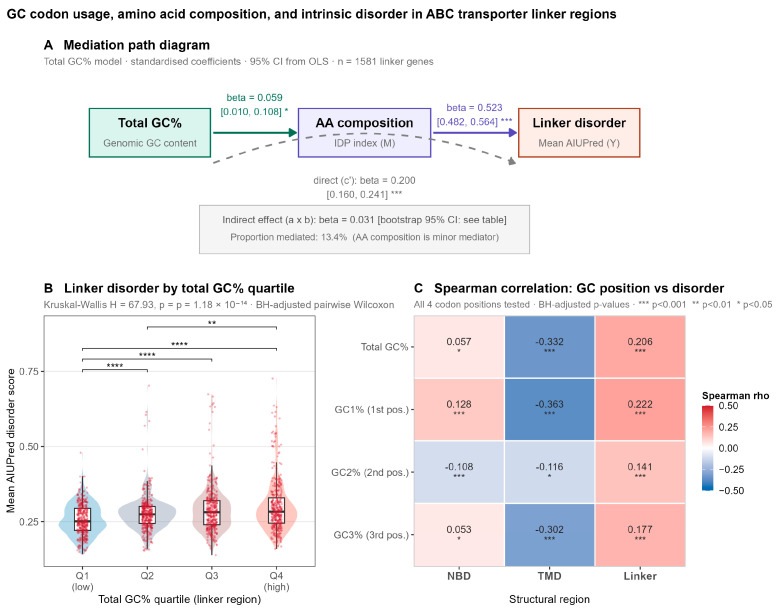
GC codon usage, amino acid composition, and intrinsic disorder in ABC transporter linker regions. (**A**) Mediation path diagram for the total-GC% model (*n* = 1581 linker genes): total genomic GC content (exposure), IDP index (mediator *M*), and mean AIUPred linker disorder (outcome *Y*); standardised β coefficients with 95% CI from OLS regression and bootstrap mediation (5000 resamples). (**B**) Mean AIUPred linker disorder score stratified by total GC% quartile; Kruskal–Wallis *H* = 67.93, *p* = 1.18 × 10^−14^; BH-adjusted pairwise Wilcoxon significance shown. **** *p* < 0.0001. (**C**) Spearman rank correlation (ρ) between GC content at each codon position (total GC%, GC1%, GC2%, GC3%; rows) and mean AIUPred disorder per structural region type (NBD, TMD, linker; columns); BH-adjusted *p*-values annotated.

To test whether the GC content–disorder association was a direct effect, independent of amino acid composition, we conducted a formal mediation analysis with the IDP amino acid index as the mediator (Figure 5). Mean linker disorder across GC% quartiles was positively associated with total GC% (Kruskal–Wallis H = 67.93, *p* = 1.18 × 10^−14^; Figure 5B). Spearman correlations between GC content and mean disorder were positive and significant for all four codon positions in linker regions (total GC%: ρ = 0.206; GC1%: ρ = 0.222; GC2%: ρ = 0.141; GC3%: ρ = 0.177; all *p* < 0.001; Figure 5C; full co-don-position breakdowns with OLS R^2^ and standardized direct effects are shown in Supplementary Figure S5), consistently negative in TMDs (total GC%: ρ = −0.332, *p* < 0.001) and weak in NBDs (total GC%: ρ = 0.057, *p* < 0.05). Mediation analysis revealed that the total GC% effect on linker disorder was not dependent on amino acid composition (c′ = 0.200, 95% CI [0.160, 0.241], *p* < 0.001; Figure 5A), while the indirect effect via the IDP amino acid index was small (ACME β = 0.031; proportion mediated 13.4%). The consistency of the direct GC effect across both non-synonymous positions (GC1%, GC2%) and synonymous wobble position (GC3%) suggest that the association reflects broader genomic GC environment rather than a codon-position specific mechanism. These analyses, along with the PGLS results, suggest that linker disorder is primarily driven by amino acid composition, while the effect of genomic GC content is secondary, statistically significant but region-specific; positive in linkers, negative in TMDs and negligible in NBDs.

### 2.3. PTM Site Distribution and Co-Localization with Disordered Regions

**Figure 6:**
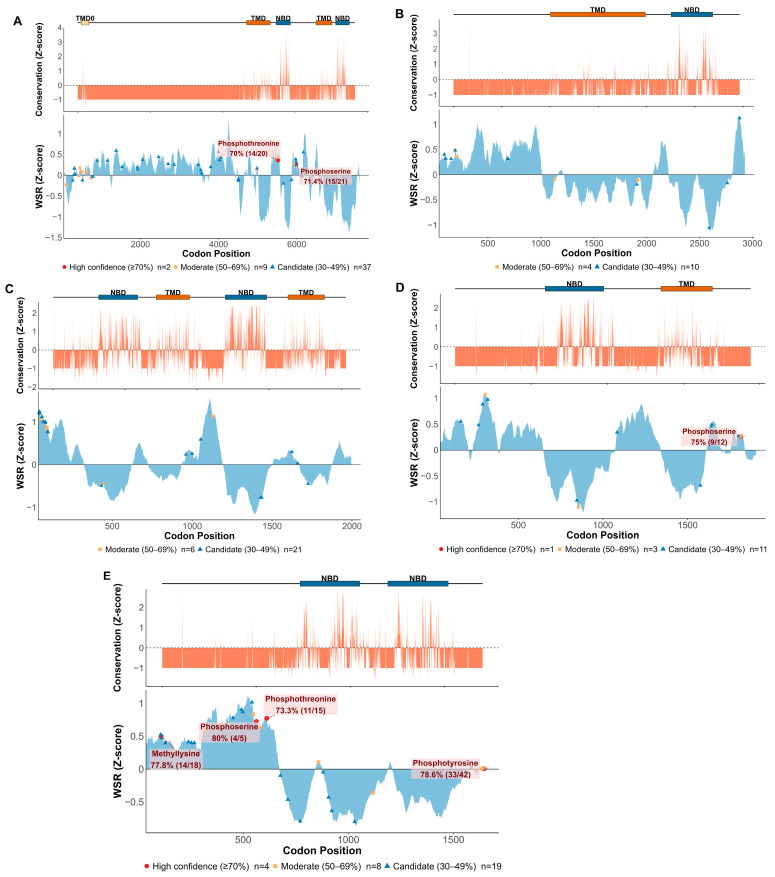
Sequence conservation and site-wise evolutionary rate with conserved PTM site annotation across ABC transporter architectural classes. For each panel, two tracks are shown along the codon alignment position (*x*-axis). Upper track: AL2CO sequence conservation Z-score; positive values indicate conservation above the alignment mean; dashed line = 0; grey shading = annotated NBD and TMD regions. Lower track: IQ-TREE2 site-wise relative evolutionary rate (WSR) Z-score overlaid with conserved predicted PTM sites from MusiteDeep (green circles; score ≥ 0.5 and cross-species conservancy ≥ 50%); PTM type labelled per site. (**A**) Full forward transporters. (**B**) Half forward transporters. (**C**) Full reverse transporters (ABCG). (**D**) Half reverse transporters. (**E**) NBD-only soluble proteins.

To assess the regulatory potential of disordered regions across ABC transporter subfamilies, predicted PTM sites from MusiteDeep were mapped onto aligned sequence positions and overlaid with disorder scores (Figure 6).

The AL2CO conservation Z-scores showed a strong asymmetric pattern over sequence alignment for all the five architectural classes. Residues within the annotated NBD segments showed the highest positive conservation (Z-scores up to ~+4 in the complete forward trans-porters; Figure 6A), corresponding to the well-known conserved Walker A, Walker B and LSGGQ signature motifs [6,7]. TMD regions also showed higher conservation compared to the non-domain regions, but less than the NBDs, reflecting greater structural diversity among the subfamilies [6]. In contrast, the non-domain regions, including the TMD0 region, the N-terminal pre-TMD0 segment and the NBD1-TMD2 inter-domain region, were dominated by negative conservation Z-scores close to −1, meaning that these regions have high sequence variability relative to the alignment average. This trend was seen for all five architectural classes and agrees with prior observations that inter-domain linkers in multidomain proteins evolve more rapidly than structured domain cores [30].

WSR Z-scores showed a partly inverse pattern to conservation. All five architectural classes of NBD regions showed negative WSR Z-scores, reflecting slow rates of evolution under strong purifying selection to preserve the NBD fold and catalytic residues. Conversely, positive WSR Z-scores were observed in non-domain and inter-domain regions, with the maximum centered outside of annotated domains. A few non-domain sites passed the +2 Z-score threshold and became fast-evolving outliers.

Mapping predicted PTM sites to the WSR landscape revealed a spatial pattern across architecture. In full forward transporters, predicted phosphoserine, phosphothreonine, methyllysine and ubiquitination sites co-localized primarily with regions with negative WSR Z-scores within the NBD1 and NBD2 blocks, but not with the most rapidly evolving non-domain positions (Figure 6A). The same pattern was also seen with half forward transporters (Figure 6C) and NBD-only proteins, where phosphothreonine, phosphoserine, methylarginine and O-linked glycosylation sites mapped to slowly evolving locations at or near the NBD cores (Figure 6D,E). In full reverse transporters, a single phosphoserine site was identified at a conserved slowly evolving position within the NBD-TMD interface (Figure 6B). Half reverse transporters displayed a broader spectrum of conserved PTM sites including phosphoserine, phosphothreonine, O-linked glycosylation, methylarginine, N6-acetyllysine, and phosphotyrosine. Several of these sites co-localized at the slow-evolving positions spanning the NBD-TMD boundary (Figure 6D). The preferential localization of predicted PTM sites to slowly evolving positions, and not at rapidly evolving non-domain regions, is not expected under a neutral model, and suggests that a subset of these sites may be maintained by stabilizing selection for their modifiable state across evolutionary timescales.

### 2.4. PTM Site Distribution, Conservancy, and Enrichment

**Figure 7:**
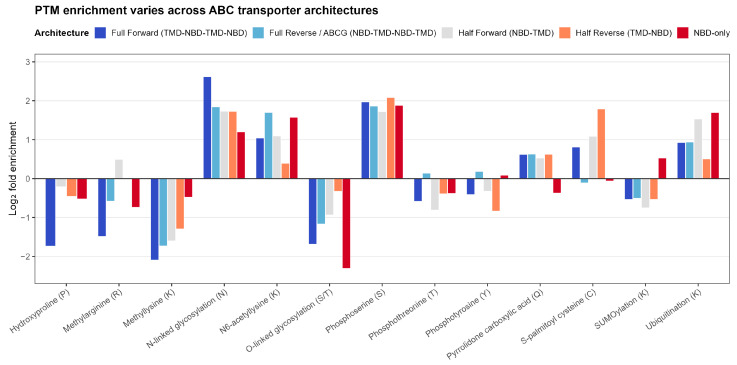
Per PTM type enrichment across ABC transporter architectural classes. Log_2_ fold enrichment (Log_2_FE) per modification type per architectural class, calculated relative to the expected rate given residue composition. Positive values indicate enrichment; negative values indicate depletion. Modification types shown: phosphoserine (pSer), phosphothreonine (pThr), phosphotyrosine (pTyr), N-linked glycosylation (N-glyc), O-linked glycosylation (O-glyc), ubiquitination (Ub), N6-acetyllysine (AcK), methylarginine (MeR), methyllysine (MeK). Bars represent mean Log_2_FE; error bars = 95% CI.

**Figure 8:**
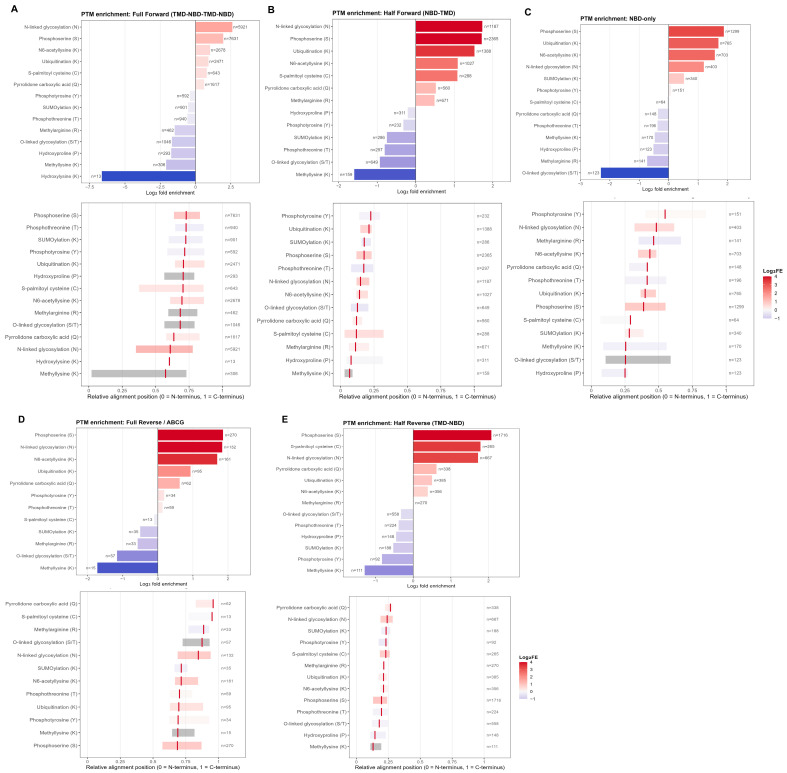
Log_2_FE PTM enrichment and distribution across architectural classes and subfamilies. Additional breakdown of PTM distribution by subfamily and region type, with conserved sites (MusiteDeep score ≥ 0.5 and cross-species conservancy ≥ 50%) highlighted. (**A**) Full forward transporters. (**B**) Half forward transporters. (**C**) NBD-only soluble proteins. (**D**) Full reverse transporters (ABCG). (**E**) Half reverse transporters.

To characterize the PTM landscape more broadly and identify sites showing cross-species conservation, predicted PTM sites were mapped across all architectural classes as a function of alignment position and scored for modification type enrichment and cross-species conservancy (Figure 7 and Figure 8).

All architectural classes showed enrichment of predicted PTM sites in disordered non-domain regions. Phosphoserine and phosphothreonine had the highest predicted frequencies in the full forward, half forward and ABCG configurations. Enrichment analysis (Figure 7) further suggested that N-linked glycosylation and phosphoserine were the most highly positively enriched (Log_2_FE) modifications, where enrichment is calculated relative to the global PTM prediction rate and corrected for target residue abundance in the combined alignment, making values comparable across modification types and architectural classes. This pattern agrees with the well-known over-representation of phosphorylation and N-glycosylation sites in disordered protein regions [31,32]. Long ABCC members exhibited abundant high predicted PTM scores in the N-terminal pre-TMD0 region, the NBD1-TMD2 linker, and the C-terminal tail beyond NBD2 for full forward transporters. In the ABCA and ABCB sequences, the predicted PTM sites had a lower density but similar positional bias towards non-domain regions.

The predicted PTM density in full reverse (ABCG) transporters was more widely distributed along the aligned length than in full forward transporters. This is probably because of the relatively smaller domain core in ABCG members, exposing a larger fraction of the total sequence to modification-permissive disordered regions [8,9]. Within the ABCG PTM landscape, we observed taxonomic variation: fungal sequences contained PTM-dense patches at different positions than plant sequences, suggesting that the positional distribution of predicted regulatory sites diverged across kingdoms within the same subfamily. The ABCG clade’s functional diversification extends ABC transporters beyond canonical drug efflux to micronutrient homeostasis; *Chlamydomonas* insertional mutagenesis identified two ABC transporter mutants with altered molybdenum sensitivity/resistance, providing genetic evidence for this expanded function [33]. The elevated PTM distribution in full reverse ABCG transporters reflects the regulatory complexity supporting this functional diversity across kingdoms.

A total of 140 predicted PTM sites satisfied a tiered conservation criterion (MusiteDeep score ≥ 0.5; cross-species conservancy ≥ 30%), stratified as 7 high-confidence sites (conservancy ≥ 70%), 33 moderate-confidence sites (conservancy 50–70%), and 100 candidate sites (conservancy 30–50%; Supplementary Table S5). The tier thresholds follow the stratified PTM conservation framework of Beltrao et al. [34] and Minguez et al. [35], in which conservation is assessed across species at increasing stringency to separate broadly conserved regulatory sites from lineage-restricted candidates. The high-confidence tier (≥70%) is robust to threshold variation, as no sites in this tier fell below 70% when the minimum occupancy filter was relaxed. High-confidence sites were identified in full forward (*n* = 2), half reverse (*n* = 1), and NBD-only (*n* = 4) architectures. Among the most conserved predictions were a phosphotyrosine site at NBD-only alignment position 1644, corresponding to ABCE1 Y594 (conservancy 78.6%, score 0.906), and a methyllysine site at position 110 (conservancy 77.8%, score 0.743). These represent previously unreported regulatory modifications in NBD-only proteins not annotated in UniProt or PhosphoSitePlus.

### 2.5. Site-Specific Selection Pressure Across Architectural Classes

**Figure 9:**
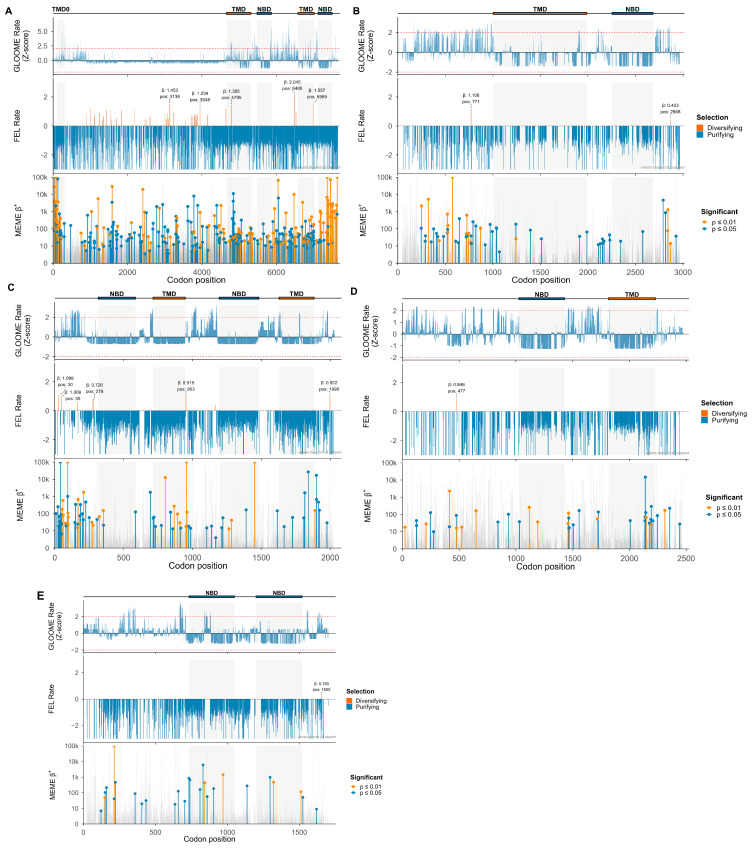
Site-specific structural state transitions and selection pressure across ABC transporter architectural classes. For each panel, three tracks are shown along the codon-alignment position (*x*-axis); grey shading denotes annotated domain regions (NBD and TMD). Top track: GLOOME-inferred structural state transition Z-score, quantifying the rate of disorder gain and loss events per site across the phylogeny; red dashed lines = ±2 Z-score threshold; sites exceeding +2 indicate structurally labile positions where disorder state has changed repeatedly across lineages. Middle track: Fixed Effects Likelihood (FEL) rate per codon position; orange bars = diversifying selection (dN > dS); blue bars = purifying selection (dN < dS); labeled sites indicate highest-β FEL-positive positions. Bottom track: MEME episodic positive selection β+ values (log scale); orange circles = *p* ≤ 0.01; blue circles = *p* ≤ 0.05; grey stems = non-significant sites. (**A**) Full forward transporters. (**B**) Half forward transporters. (**C**) Full reverse transporters (full-size ABCG/PDR). (**D**) Half reverse transporters. (**E**) NBD-only soluble proteins.

FEL analysis indicated a pervasive purifying selection across the five architectural classes, with the strongest purifying signal concentrated in the annotated NBD regions (Figure 9). This pattern, consistent with functional constraints on ATP-binding and hydrolysis residues, was seen across architectures and would be expected given the well-documented conservation of Walker A, Walker B, and LSGGQ motifs [6]. Fewer sites had positive FEL estimates (diversifying selection), but the total number was small (0–41 sites per class; median = 3), limiting statistical power for enrichment analyses in most architectures. For full forward transporters, FEL-positive sites were mainly distributed in non-domain regions, with only two FEL-positive sites in the TMD region (Figure 9A). In full reverse transporters, FEL-positive sites were concentrated in the N-terminal pre-NBD region and the NBD–TMD interface (Figure 9C).

Half forward transporters (525, 931, 972, Figure 9B) contained three FEL-positive sites, all located in non-domain regions flanking the TMD. Half reverse transporters had one FEL-positive site (position 477, β = 0.846; Figure 9D) whereas NBD-only proteins had a minimal diversifying signal (one site, position 1655, β = 0.141; Figure 9E), which is consistent with the strong functional constraint in NBD, as this group’s main component. A sample number may, however, affect HyPhy, especially FEL, thereby altering the tool’s sensitivity. 

MEME analysis identified episodic positive selection in a larger number of sites than FEL in all architectural classes, consistent with MEME being more sensitive to detect selection acting only in a subset of lineages rather than across the full tree [36]. The highest β+ values were found at non-domain positions, with several sites showing β+ > 100 and a subset approaching 10^4^–10^5^ in both full forward and full reverse transporters, suggesting that rare but highly accelerated substitutions happened in specific lineages. In full forward transporters, significant MEME sites (*p* ≤ 0.01) were enriched in the N-terminal pre-TMD0 region and at positions adjacent to NBD1 and NBD2 (Figure 9A). In full reverse transporters, MEME sites were distributed widely over both domain-flanking and non-domain positions, with the highest β+ values in the N-terminal pre-NBD region (Figure 9C). In NBD-only proteins, MEME-positive sites were primarily found in the N-terminal flank and inter-NBD linker, surrounding rather than being within the conserved NBD cores (Figure 9E).

To formally test the preference of sites under selection to be in non-domain regions, we performed Fisher’s exact tests to compare the proportion of sites under selection in non-domain and annotated domain regions for each architectural class (Supplementary Figure S6). The diversifying selection detected by FEL was significantly non-domain enriched in full reverse transporters (7/7 diversifying sites in non-domain regions; OR = ∞, BH-adjusted *p* = 0.013) and full forward transporters (37/41 in non-domain regions; OR = 6.93, adjusted *p* = 0.002). For MEME detected episodic selection, full reverse (OR = 2.32, adjusted *p* = 0.001) and half forward (OR = 3.99, adjusted *p* < 0.001) transporters had significant non-domain enrichment, while full forward transporters had significant enrichment of episodic sites within domain cores (OR = 0.52, adjusted *p* < 0.001), suggesting that in this large multidomain architecture, episodic adaptive substitutions also target structured domains. Interestingly, half reverse and NBD-only transporters were not significantly enriched in either direction. Together, these findings provide evidence that the evolutionary landscape of ABC transporters is shaped by pervasive purifying selection within the domain cores, with episodic diversification showing architecture-dependent non-domain enrichment rather than a uniform superfamily-wide trend.

GLOOME-inferred structural state transition profiles (Figure 9, top tracks) revealed that disorder gain and loss events were unevenly distributed across the alignment in all five classes. Domain cores (NBD and TMD) showed compressed Z-scores near zero, indicating stable structural states maintained across lineages, while non-domain regions exhibited broader variance with multiple positions exceeding the +2 threshold, indicating sites where disorder state has been gained or lost repeatedly across the phylogeny. These structurally labile positions were concentrated at domain boundaries and in linker segments, consistent with the interpretation that non-domain regions undergo structural remodeling while domain cores maintain a stable ordered state.

## 3. Discussion

### 3.1. Non-Domain Regions Are Structurally and Evolutionarily Distinct from Domain Cores

The key finding of this study is that non-domain regions of ABC transporters, including inter-domain linkers, N-terminal flanks, and C-terminal extensions, are not passive structural connectors but have distinct structural, compositional, and evolutionary signatures that vary systematically across architectural classes. Linker disorder exhibited significantly greater levels than NBDs and TMDs across all five architectures (*p* < 0.0001, BH-adjusted Wilcoxon), and this hierarchy was robust after phylogenetic correction (Pagel’s λ = 0.955–0.972 across PGLS models), indicating that the pattern observed reflects true evolutionary constraint rather than an artifact of phylogenetic non-independence. These results are in agreement with the general view that IDRs in other protein families are functional elements whose properties are maintained by selection [18,22]. The lower disorder of TMD cores reflects the physicochemical constraints of the lipid bilayer, which favor hydrophobic, order-promoting residues. However, three observations argue that physicochemistry alone cannot fully explain the observed patterns. First, PGLS M2 identifies a composition-independent GC–disorder association (ΔR^2^ = 0.016–0.037; Table 1). Second, GLOOME analysis reveals repeated disorder gain and loss events at domain boundaries across lineages (Figure 9), inconsistent with a static physicochemical property. Third, the three full subfamilies show dramatically different L2 disorder despite sharing the same membrane environment (ABCA median 0.083 vs. ABCC median 0.526; Supplementary Figure S5). Thus, linker disorder is a phylogenetically conserved trait shaped by both physicochemical constraints and subfamily-specific evolutionary pressures [21].

Linker disorder shows a significant phylogenetic signal and is informative. This implies that closely related sequences tend to share similar linker disorder profiles, a pattern inconsistent with neutral evolution of linker composition. The high λ values for all three PGLS models indicate that the amino acid identity of the linker regions has been inherited and conserved along lineages in a manner consistent with Brownian motion under stabilizing constraint [37,38]. This is in line with observations for other IDR-containing proteins, where functional properties are conserved at the level of biophysical features rather than primary sequence [21,22]. Whether the specific linker regions identified here perform similar regulatory functions in ABC transporters, as has been shown for the CFTR R-domain and MRP1 L0 linker, remains to be experimentally validated for the wider superfamily.

It is important to point out that the disorder predictions shown here are based on a single algorithm (AIUPred) [39]. AIUPred combines energy estimates with deep learning and was validated against NetSurfP-3.0 secondary structure predictions [40] (Supplementary Figure S1). Consensus approaches that consider multiple disorder predictors may yield quantitatively different estimates of the disorder fraction for individual sequences.

### 3.2. L2 Emerges as the Primary Regulatory Linker in Full Forward Transporters

In full forward transporters, the L2 linker between NBD1 and TMD2 had the highest median length (176 aa) and the highest median disorder fraction (0.38) among all inter-domain segments, whereas L1, L3, and Cflank were largely ordered. This positional specificity is consistent with experimental evidence: L2 corresponds to the regulatory (R) domain of CFTR (ABCC7) and related ABCC family members, where extensive phosphorylation of disordered serine residues modulates NBD dimerization and channel gating [12,23]. Recent cryo-EM structures of phosphorylated Ycf1p, a yeast ABCC homolog, directly resolved the L2/R-domain bridging NBD1 and NBD2 in a transport-competent conformation, demonstrating that its disordered character is functionally coupled to the conformational transport cycle [23]. Our computational results generalize this observation from a single well-studied transporter to the entire class of forward transporters, indicating that increased L2 disorder and PTM enrichment are general organizational principles of ABCA, ABCB, and ABCC transporters, not a unique property of CFTR. The co-localization of predicted phosphoserine, phosphothreonine, ubiquitination, and methyllysine sites with slowly evolving positions in NBD1 and NBD2 further supports the notion that a subset of these regulatory sites is maintained by stabilizing selection, consistent with the constitutive phosphorylation of ABCA1 at Ser2054, required for cholesterol efflux activity [17,25,39].

### 3.3. Genomic GC Content Influences Linker Disorder Primarily Through Amino Acid Composition

PGLS modeling across all four codon positions shows that the GC–disorder association in ABC transporter linker regions is driven mainly by non-synonymous sites. The ranking of GC predictors reveals a clear mechanistic pattern: GC2% (second codon position) was the strongest predictor with an adjusted R^2^ of 0.164, followed by GC1% (adj. R^2^ = 0.081), total GC% (adj. R^2^ = 0.072), and GC3% at the synonymous wobble position (adj. R^2^ = 0.036). This ranking directly reflects codon table mechanics—at the second position, U codes for hydrophobic residues (Leu, Ile, Val, Phe, Met) while C or G encode polar, disorder-promoting residues (Ala, Pro, Arg, Gly) [41]. Since the first and second codon positions determine amino acid identity, high GC at these sites inherently biases the proteome toward disorder [28,29]. Yet despite this clear mechanistic foundation, GC2% captures information about disorder that extends beyond these physicochemical properties. Adding GC2% to amino acid composition significantly improved predictions (M2 versus M3, ΔAIC = −95.6, ΔR^2^ = 0.036), indicating that the GC2% signal carries additional information not accounted for by composition alone. The relationship further reveals regional specificity, with positive associations in linkers but negative associations in transmembrane domains. This sign reversal is inconsistent with a uniform physicochemical mechanism and instead suggests region-specific evolutionary pressures exploiting the same encoding pathway in opposite directions (Figure 5C). Amino acid composition alone explained 39.1% of variance in linker disorder (M3; Table 1), confirming it as the primary route through which GC influences disorder, as suggested by Peng et al. [28] and Basile et al. [29].

However, all four GC predictors remained statistically significant partial effects after accounting for amino acid composition (all *p* < 0.001 in M2 models; Table 1), and the integrated model (GC2% + AA composition PCs; adj. R^2^ = 0.426, AIC = −6554.1) explained a much larger amount of variance than amino acid composition alone (ΔAIC = −95.6 compared to M3). The residual GC effect after controlling for composition (ΔR^2^ = 0.016–0.037 across different predictor variants) supports the mediation analysis, which showed partial mediation (total GC% proportion mediated 13.4%; direct effect c′ = 0.200, *p* < 0.001). The GC-disorder relation was highly region-specific across all four codon positions: positive in linker regions, negative in TMDs, and negligible in NBDs, indicating that most genome-wide studies that combine region types would greatly mask this relationship. The composition-independent residual GC effect (ΔR^2^ = 0.016–0.037) may reflect several non-mutually exclusive mechanisms beyond amino acid identity, such as the influence of mRNA secondary structure on co-translational folding rates [27], codon usage bias affecting translational speed and nascent-chain dynamics, and genome-wide mutational pressure creating correlated variation across linked loci [42]. Disentangling these contributions will require experimental approaches such as synonymous codon substitution or ribosome profiling, which are beyond the scope of the present computational analysis.

### 3.4. Domain Boundaries Are the Sites of Structural State Lability

Structural transition rates inferred from GLOOME, which measure disorder-to-order gain and loss events over lineages, were maximal at domain boundaries, not in linker cores. This result challenges the idea that linker regions are the primary sites of structural evolutionary change. Instead, the data indicates that linker cores remain in a relatively stable disordered state, while regions at the interface between structured domains and flanking disordered segments experience more frequent structural remodeling. This pattern is consistent with the concept of molecular recognition features (MoRFs), short segments within IDRs that can fold in concert upon binding to interaction partners [18,43]. Domain boundaries in ABC transporters may therefore serve as evolutionarily labile MoRF-like elements whose structural state is under lineage-specific selection, whereas the majority of linker regions retain a stable disordered character. This interpretation is in agreement with structural evidence indicating that the intracellular coupling helices at the TMD–NBD interface of ABCB and ABCC transporters undergo substantial conformational reorganization during the transport cycle [2]. A caveat is that alignment-dependent analyses that underpin these observations (GLOOME, FEL, MEME, AL2CO) are sensitive to alignment quality, and that highly divergent non-domain regions may be poorly aligned even after ClipKIT trimming, potentially inflating estimates of evolutionary rate at boundary positions. Independent validation using structure-aware alignment methods or pairwise dN/dS approaches would help confirm these patterns. The concentration of structural state transitions (GLOOME) at domain boundaries contrasts with the results of formal selection enrichment testing (Fisher’s exact test), which showed that episodic positive selection (MEME) is enriched in non-domain regions as a whole rather than specifically at boundary positions and that this enrichment is architecture-dependent (significant in full reverse and half forward but not in half reverse or NBD-only transporters; Supplementary Figures S4 and S6). This distinction suggests that boundary regions undergo structural remodeling (disorder gain and loss across lineages) without necessarily accumulating the amino acid substitutions that would be detected as positive selection by codon-based methods.

### 3.5. PTM Distribution Reflects Both Disorder Enrichment and Positional Conservation

Predicted PTM sites were enriched in disordered non-domain segments among all architectural classes, with phosphoserine and phosphothreonine dominating full forward, half forward, and ABCG configurations. This enrichment is consistent with the well-established over-representation of phosphorylation sites in IDRs [31,32] and with experimental phosphoproteomic data from CFTR, ABCA1, MRP1, and Ycf1p showing that functionally important phosphorylation events occur in disordered linker regions [12,13,23]. The surprising result was that many of the predicted PTM sites co-localized with slowly evolving sites in NBD cores where purifying selection is strong. This implies that a subset of regulatory modification sites is evolutionarily conserved and maintained by stabilizing selection, unlike the general population of PTM sites in fast-evolving non-domain regions. A comparable two-level arrangement of PTM sites, with regulatory residues that are constitutively conserved embedded in faster-evolving disordered backgrounds, has been suggested for other multidomain signaling proteins [22,44]. The presence of O-linked glycosylation, methylarginine, and N6-acetyllysine sites in the half-forward and NBD-only classes expands the predicted regulatory PTM landscape beyond phosphorylation in these less-studied architectural classes and warrants experimental validation. All PTM sites reported here are predictions from MusiteDeep and cross-referenced to experimentally annotated sites in UniProt Swiss-Prot for human ABC transporters, confirming that most of the available reference sites were recovered, although experimental PTM coverage in UniProt is sparse for most subfamilies and concentrated in well-characterized members such as CFTR and ABCG1 (Supplementary Table S7). Functional claims require experimental validation, by phosphoproteomics or site-directed mutagenesis, of novel candidate sites, especially those found in non-human sequences or in previously unstudied architectural classes.

### 3.6. Implications for Linker-Targeted Drug Discovery

Non-domain regions of ABC transporters represent a largely underexplored space for functional modulation. Current pharmacological strategies predominantly target the structurally well-characterized NBD and TMD cores through ATP-competitive or substrate-competitive inhibition, which raises selectivity concerns given the high conservation of these sites across family members. The disordered, PTM-enriched linker regions identified here offer a mechanistically distinct alternative: these segments regulate transporter activity through phosphorylation-dependent conformational changes and protein–protein interactions rather than through direct catalysis and are therefore amenable to modulation that does not compete with substrates or nucleotides.

That such regulation is functionally consequential is well established in specific ABC transporters, though whether the mechanisms described below generalize across all subfamilies remains to be tested. Linker mutations in the CFTR R-domain and the MRP1 L0 linker cause disease by disrupting phosphorylation-dependent gating [12,13,14], demonstrating that these segments are essential regulatory elements. The clinical success of CFTR modulators further illustrates the therapeutic potential of inter-domain communication: the corrector VX-809 (lumacaftor) acts on transmembrane domain 1 and propagates allosteric stabilization to the NBD1 interface, rescuing folding defects caused by the F508del mutation [45]. This allosteric principle extends beyond CFTR—extracellular non-domain mutations in MRP1 couple to transmembrane conformational changes [46], establishing that perturbations in non-domain segments can propagate functional effects across the full transporter architecture. More recently, Heinkel et al. [47] demonstrated that the disordered, phosphorene-rich linker of the *M. tuberculosis* transporter Rv1747 undergoes phosphorylation-dependent liquid–liquid phase separation, regulated by multiple Ser/Thr kinases, suggesting that condensate formation in non-domain regions may represent a regulatory mechanism in bacterial ABC transporters. While CFTR and MRP1 are the best-characterized examples, the patterns reported here suggest regulatory linker function extends across the superfamily. Elevated L2 disorder in all three full-forward subfamilies (ABCA, ABCB, ABCC), conserved phosphosites at slowly evolving positions, and architecture-dependent selection enrichment collectively indicate that non-domain regulation is a general feature of ABC transporters. Whether phosphorylation-dependent gating operates broadly, or whether other regulatory modes (e.g., protein–protein interactions, condensate formation) predominate, remains to be determined. These findings also have therapeutic implications. Computational strategies for targeting disordered regions include fragment-based mapping to identify transient pockets in disordered ensembles [48], modulation of biomolecular condensate formation [49], and the design of conformationally adaptive peptides matching dynamic disordered targets [50], suggesting that ABC transporter linkers may represent underexplored therapeutic targets. These conclusions should, however, be interpreted in light of dataset limitations. The taxonomic composition is weighted toward mammals (~40%), and some architectural classes remain underrepresented (full reverse, *n* = 69), constraining the statistical power of class-specific analyses and potentially missing linker diversity in under-sampled lineages. The disorder and PTM maps presented here therefore serve as a prioritization resource for future experimental and structural studies rather than definitive evidence of regulatory function or therapeutic druggability.

## 4. Materials and Methods

### 4.1. Sequence Retrieval, Quality Filtering, and Architecture Classification

A total of 1581 ABC transporter protein sequences were retrieved from the KEGG database [51] using a custom Python script interfacing with the KEGG REST API, and querying by KEGG organism codes. The database included prokaryotes (*E. coli*, *M. tuberculosis*, *P. aeruginosa*, *V. cholerae*, *S. aureus*), fungi (*S. cerevisiae*, *C. albicans*), plants (*A. thaliana*, *Z. mays*, *O. sativa*), invertebrates (*D. melanogaster*, *C. elegans*), and vertebrates across five classes. Exact duplicate sequences were removed using SeqKit v2.8.0 rmdup --by-seq [52], and sequences containing non-standard residues or truncated open reading frames were filtered using HyPhy v2.5 [53]. Domain boundaries were annotated using HMMER v3.4 against Pfam v36.0 [54,55], searching for ABC_tran (NBD), ABC_membrane, ABC_membrane_2, ABC2_membrane, ABC2_membrane_3, and ABC2_membrane_7 profiles at E-value ≤ 1 × 10^−3^. A custom Python script extracted domain boundary coordinates and classified sequences into five architectural classes based on domain count and order, following established structural taxonomy of Wilkens [26] and Dean & Annilo [1]: Full_Forward (TMD–NBD–TMD–NBD; *n* = 751), Full_Reverse (NBD–TMD–NBD–TMD, ABCG; *n* = 69), Half_Forward (TMD–NBD; *n* = 372), Half_Reverse (NBD–TMD; *n* = 228), and NBD_only (NBD–NBD, ABCE/ABCF; *n* = 154). The full reverse class comprises plant (*n* = 53), fungal (*n* = 9), and protist (*n* = 6) sequences corresponding to full-size ABCG transporters of the pleiotropic drug resistance (PDR) type, which are expressed as single-chain NBD–TMD–NBD–TMD polypeptides [56,57,58,59]. Structural regions were defined as N-terminal flank (Nflank), inter-domain linkers (L1–L3, minimum gap ≥ 10 residues), and C-terminal flank (Cflank). Organism information and KEGG organism codes are provided in Table S1. Domain composition per sequence is visualised in Figure 1 (main panel) as horizontal bars mapped onto trimmed alignment coordinates, with ABC_membrane family domains shown in orange/red tones and ABC_tran (NBD) domains in blue.

### 4.2. Sequence Alignment, Phylogenetic Inference, and Transmembrane Topology

Protein sequences per architectural class were aligned using MAFFT v7.520 L-INS-i [60] and trimmed with ClipKIT v1.3.0 kpic-smart-gap mode [61]. Codon-aware nucleotide alignments were generated using Pal2Nal [62]. Phylogenetic trees were inferred with IQ-TREE2 using ModelFinder Plus (MFP) for automatic model selection and ultrafast bootstrap approximation (1000 replicates) [63,64]. Trees served as input for downstream analyses, and for ordering sequences in heatmap visualizations. Transmembrane topology was predicted per sequence using DeepTMHMM v1.0.24 [65] and validated with TOPCONS [66]. Consensus TM frequency profiles were constructed by mapping predicted TM positions onto alignment coordinates and computing positional occupancy frequencies. These profiles are shown in Figure 1 (bottom right panel) for each architectural class: the upper trace represents DeepTMHMM predictions and the lower trace TOPCONS predictions; blue indicates cytoplasmic loop frequency, grey indicates extracellular loop frequency, and red shading marks positions with >50% transmembrane helix occupancy across sequences in the class.

### 4.3. Structural Characterisation: Disorder, Secondary Structure, and Region-Specific Analysis

Per-residue intrinsic disorder scores were predicted using AIUPred [39], which integrates energy estimation with deep learning (score range 0–1; threshold 0.5 for disorder classification). Secondary structure propensities (helix, sheet, coil) were predicted using NetSurfP-3.0 [40]. All predictions were performed on unaligned sequences and subsequently mapped onto trimmed alignment coordinates per architectural class for heatmap visualization. Heatmaps were constructed in R v4.4.2 using a custom pipeline with ggtree, ggplot2, and patchwork [67,68]. Disorder fraction and region length distributions were compared using Kruskal–Wallis tests with Benjamini–Hochberg-corrected pairwise Wilcoxon tests (rstatix package v0.7.3). Cross-architecture comparisons were performed using Mann–Whitney U tests with Cliff’s delta as a non-parametric effect size measure.

### 4.4. Post-Translational Modification Prediction and Enrichment

PTM sites were predicted across all sequences using MusiteDeep [69], a deep learning framework trained on experimentally verified data from UniProt and PhosphoSitePlus. Predictions were generated for 14 modification types, including phosphoserine, phosphothreonine, phosphotyrosine, N6-acetyllysine, methylarginine, methyllysine, N-linked and O-linked glycosylation, S-palmitoyl cysteine, SUMOylation, ubiquitination, hydroxyproline, hydroxylysine, and pyrrolidone carboxylic acid. Sites were retained at score ≥ 0.5 and verified for chemical validity (residue–modification pairing).

Conservancy (%) = (*n*_passing_/*n*_with-residue_) × 100

where *n*_passing_ is the number of sequences with a chemically valid prediction scoring ≥ 0.5 at that column and *n*_with-residue_ is the number of sequences carrying a non-gap residue, following the gap-correction principle of Valdar [70] and Capra & Singh [71]. Three filters were applied before tier assignment: (i) *n*_with-residue_ ≥ max (5, ⌈0.02 × *n*_matched_⌉), where *n*_matched_ is the number of sequences present in both the alignment and the MusiteDeep output; (ii) *n*_passing_ ≥ 3; and (iii) the score and chemical-validity filters above. Sites passing all filters were stratified into three tiers: high-confidence (≥70%), moderate-confidence (50–69%), and candidate (30–49%), following the stratified PTM conservation approach of Minguez et al. [35].

Log_2_ fold enrichment (Log_2_FE) per modification type per architectural class was calculated as:

Log_2_FE(t) = *log*_2_([*n_sites*(*t*)/*n_target*(*t*)]/[*n_total_sites*/*n_total_residues*])

where *n_target(t)* represents the number of chemically eligible residues across all sequences in the architecture. Conserved PTM sites were identified by applying the three-filter system and tiered stratification described above, with high-confidence sites requiring conservancy ≥ 70%. Computational predictions were benchmarked against 27 experimentally verified PTM annotations from UniProt’s Swiss-Prot for 47 human ABC transporters, with a positional tolerance of ±2 residues (Supplementary Table S7).

### 4.5. Amino Acid Composition, IDP Index, GC Content, and Mediation Analysis

Per-gene amino acid composition was computed as the fractional residue frequency for each structural region. A net intrinsic disorder propensity (IDP) index was derived as:

*IDP index* = *Σf*(*disorder-promoting*) − *Σ f*(*disorder-inhibiting*)

where disorder-promoting residues were {E, K, R, S, Q, D, P, G, T, N} and disorder-inhibiting residues were {I, L, V, F, W, Y, C, M}, following Dunker et al. [72] and Uversky et al. [73]. Architecture-specific compositional deviations (Δmean) were assessed using per-cell permutation testing (10,000 iterations, Benjamini–Hochberg FDR across 100 cells). GC content was calculated per gene per region at each codon position (total GC%, GC1%, GC2%, GC3%) and correlated with mean AIUPred disorder using Spearman rank correlation per region type. Causal mediation analysis was conducted on both canonical and non-canonical regions (*n* = 1581 genes) with GC% as the predictor, IDP index as the mediator, and mean AIUPred disorder as the outcome. The analysis followed the Baron and Kenny stepwise regression (mediation package v4.5.0, 5000 bootstrap resamples) [74] and confirmed by structural equation modelling in lavaan v0.6-21 [75] (1000 bootstrap resamples; model fit assessed by CFI, RMSEA, SRMR).

### 4.6. Phylogenetic Generalized Least Squares

To account for phylogenetic non-independence, three PGLS models were fitted using the caper R package v1.0.4 [76] with Pagel’s λ optimized by maximum likelihood [37]. Three model families were evaluated for each of four GC predictors (total GC%, GC1%, GC2%, GC3%): M1 (GC alone), M2 (GC + amino acid composition principal components), and M3 (composition PCs only, providing a baseline for ΔAIC and ΔR^2^ assessment). The outcome was mean AIUPred linker disorder (*n* = 1581). Pagel’s λ ranged from 0.955 to 0.972 across all models, confirming strong phylogenetic structuring of linker disorder. GC2% was the strongest single predictor (M1: adj. R^2^ = 0.164) and yielded the best combined model (M2: adj. R^2^ = 0.426, AIC = −6554.1). Full results for all twelve model fits are reported in Table 1.

### 4.7. GLOOME Structural State Transition Analysis

Per-site binary disorder-state assignments (0 = ordered, 1 = disordered; AIUPred threshold 0.5; gaps encoded as?) were formatted as phyletic-pattern FASTA files per architectural class. GLOOME (gainLoss executable) [77] was applied with IQ-TREE2 phylogenies supplied in Newick format. Analyses used a mixture model for gain and loss rates (_gainLossDist 1), GENERAL_GAMMA_PLUS_INV distributions for gain and loss (_gainDistributionType, _lossDistributionType), three categories each, joint maximum-likelihood optimization of parameters and branch lengths (_performOptimizations 1, _performOptimizationsBBL 1), at the mid-optimization level. Site-specific gain/loss expectations from PosteriorExpectationOfChange.txt were standardized to Z-scores; sites with Z > +2 were classified as structurally labile.

### 4.8. Site-Specific Selection and Sequence Conservation

Codon-level selection analyses were conducted using HyPhy v2.5 [53] on Pal2Nal codon alignments with IQ-TREE2 phylogenies. Site-specific dN/dS ratios were estimated using Fixed Effects Likelihood (FEL) under the MG94 × GTR substitution model with synonymous rate variation [78]. Sites classified as diversifying (dN > dS) or purifying (dN < dS) at *p* ≤ 0.05. Episodic positive selection was detected using MEME [36] (*p* ≤ 0.05; highly significant sites at *p* ≤ 0.01). Sequence conservation was quantified per site using AL2CO v1.0 [79], standardized to Z-scores.

### 4.9. Software and Statistical Environment

All analyses were performed in R v4.4.2. Key packages: tidyverse v2.0.0, ggplot2 v4.0.0, ggtree v3.14.0, patchwork v1.3.2, rstatix v0.7.3, lavaan v0.6-21, mediation v4.5.0, caper v1.0.4, RColorBrewer v1.1.3. Python v3.12.7 analyses used pandas. Sequence processing used SeqKit v2.8.0 and ClipKIT v1.3.0. Figures were exported at 300 dpi (supplementary) and 600 dpi (main figure).

## 5. Conclusions

Our characterization of intrinsic disorder, post-translational modifications, and evolutionary pressure across all five ABC transporter architectures yields four conclusions. First, linkers and flanks are consistently more disordered than cores; striking L2 divergence among ABCA, ABCB, and ABCC subfamilies (λ ≈ 0.97) reflects subfamily-specific evolutionary pressures and membrane physicochemical constraints. Second, GC content in the second codon position is most predictive of linker disorder, via encoding of hydrophobic and polar amino acids; a residual composition-independent effect (ΔR^2^ = 0.036) suggests mRNA structural or codon usage mechanisms. Third, 140 predicted PTM sites meet tiered cross-species conservation criteria, are enriched in domain-proximal and disordered regions, and await experimental validation. Fourth, episodic positive selection is enriched in non-domain regions of full-backward and half-forward transporters, but concentrated in cores of full-forward transporters, indicating architecture-dependent rather than superfamily-wide selection patterns. These results provide a framework for experimental validation and establish non-canonical regions as structurally dynamic and evolutionarily active components of ABC transporter architecture.

## Figures and Tables

**Table 1:** Phylogenetic generalized least squares (PGLS) models of GC content and amino acid composition as predictors of linker intrinsic disorder in ABC transporter genes.

GC Predictor	n	λ ᵃ	R^2^	adj. R^2^	AIC	β (GC)	SE	t	*p*-Value
**M1: GC% only—direct effect of GC content on linker disorder (*n* = 1581)**									
Total GC%	1581	0.955	0.073	0.072	−5800.5	0.0024	0.0002	11.13	<2 × 10^−16^ ***
GC1% (1st position)	1581	0.958	0.082	0.081	−5816.6	0.0038	0.0003	11.84	<2 × 10^−16^ ***
**GC2% (2nd position)**	**1581**	**0.963**	**0.164**	**0.164**	**−5966.3**	**0.0078**	**0.0004**	**17.62**	<2 × 10^−16^ ***
GC3% (3rd position)	1581	0.955	0.037	0.036	−5740.2	0.0008	0.0001	7.75	1.6 × 10^−14^ ***
**M2: GC% + AA composition—residual direct GC effect after controlling for AA composition (*n* = 1581)**									
Total GC%	1581	0.968	0.413	0.412	−6519.2	0.0015	—	—	1.8 × 10^−15^ ***
GC1% (1st position)	1581	0.969	0.407	0.406	−6503.8	0.0019	—	—	5.2 × 10^−12^ ***
**GC2% (2nd position)**	**1581**	**0.971**	**0.427**	**0.426**	**−6554.1**	**0.0040**	**—**	**—**	<2 × 10^−16^ ***
GC3% (3rd position)	1581	0.968	0.405	0.404	−6499.1	0.0005	—	—	4.7 × 10^−11^ ***
**M3: AA composition only—baseline model without GC predictor (*n* = 1581)**									
AA composition (IDP index) ᵇ	1581	0.972	0.391	0.390	−6458.5	—	—	—	—

Outcome variable: mean AIUPred disorder score of ABC transporter linker regions; *n* = 1581 genes. ᵃ λ: Pagel’s lambda estimated by maximum likelihood. λ = 1 indicates complete phylogenetic signal (Brownian motion); λ = 0 indicates phylogenetic independence. All models returned λ = 0.955–0.972. ᵇ M3 contains only amino acid composition (IDP index) as a predictor; β, SE, t, and *p* are not applicable (—). M3 provides the baseline R^2^ against which the residual direct effect of GC% in M2 can be assessed (ΔR^2^(M2 vs. M3) = +0.016–0.037). SE and t-statistic available for M1 only; — indicates not extracted for M2. R^2^ values reflect phylogenetically corrected residual variance and are not directly comparable to OLS R^2^. *** *p* < 0.001.

## Data Availability

The original contributions presented in this study are included in the article/Supplementary Material. Further inquiries can be directed at the corresponding author.

## Supplementary Materials

The following supporting information can be downloaded at: https://www.mdpi.com/article/10.3390/ijms27114699/s1.

## Abbreviations

The following abbreviations are used in this manuscript:

ABC	ATP-Binding Cassette
IDR	Intrinsically Disordered Region
NBD	Nucleotide-Binding Domain
PDR	Pleiotropic Drug Resistance
PGLS	Phylogenetic Generalized Least Squares
PTM	Post-translational Modification
TMD	Transmembrane Domain
