# Supplementary material for: Reading Between the ABCs: Intrinsic Disorder and Evolutionary Dynamics of Non-Canonical Regions in ABC Transporters
Source: Int J Mol Sci. 2026 May 23;27(11):4699. doi: 10.3390/ijms27114699 (PMC13257379; doi:10.3390/ijms27114699)
Supplement: Supplementary file 1 [file ijms-27-04699-s001.zip › ijms-4304076-supplementary.pdf]

Reading Between the ABCs: Intrinsic Disorder and Evolutionary Dynamics of Non-Canonical Regions in ABC Transporters

Ichda Arini Dinana, Yukihiko Kubota, Masahiro Ito

Supplementary Materials

Contents: Figures S1–S6; Tables S1–S7

Supplementary Figures

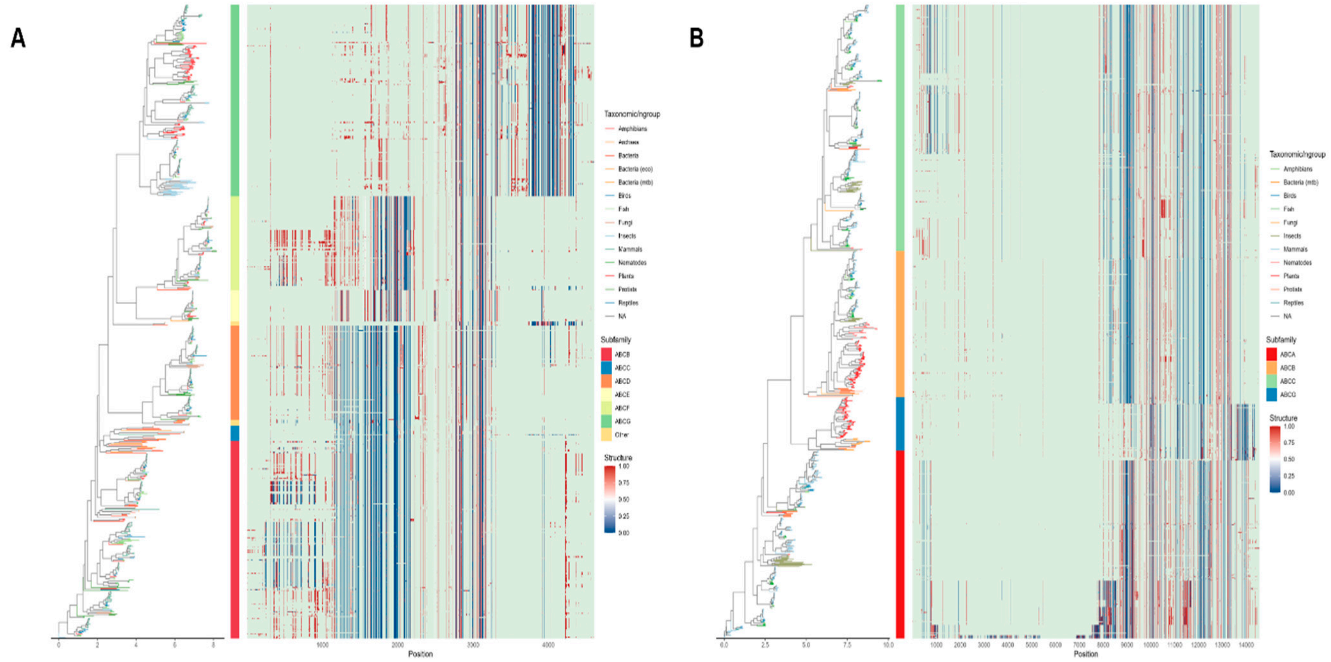

Figure S1. NetSurfP-3.0 secondary structure propensity profiles across ABC transporter architectural classes. Phylogenetically ordered heatmaps of per-position secondary structure propensity (score range 0–1; red = high helix/sheet propensity; blue = coil/disordered propensity). Domain annotation track (top): TMD regions (orange) and NBD regions (blue). Annotated domain cores show high structural propensity; inter-domain linker and flanking positions are coil-dominant across all architectural classes, corroborating AIUPred disorder predictions (Figure 2, main text). (a) Full forward and full reverse transporters (~14,000 alignment positions). (b) Half forward, half reverse, and NBD-only transporters (~4,500 alignment positions).

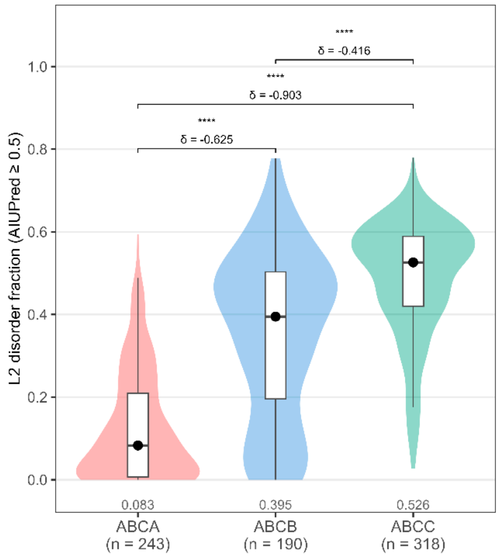

Figure S2. L2 linker disorder fraction by subfamily within full forward transporters. Violin plots with

embedded box plots; black dot = median; median values annotated below each group. Significance brackets show BH-adjusted pairwise Wilcoxon *p*-values (**** $p < 0.0001$) with Cliff's δ effect sizes. ABCC members (median 0.526) show significantly higher L2 disorder than ABCB (0.395) and ABCA (0.083), consistent with the regulatory R-domain function characterized in CFTR and related ABCC members.

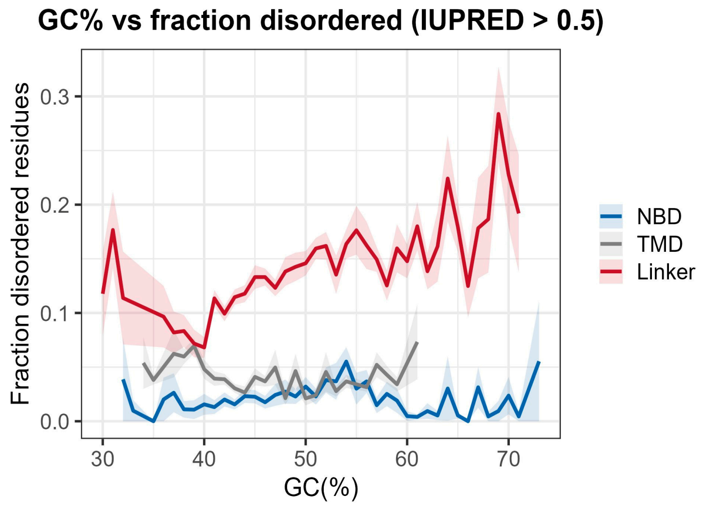

Figure S3. GC content versus fraction of disordered residues (AIUPred score > 0.5) by region type. Binned fraction of residues exceeding the 0.5 disorder threshold as a function of total coding sequence GC content (30–75%), stratified by region type (NBD, blue; TMD, grey; linker, red); shaded bands = 95% CI; data pooled across all five architectural classes. The positive GC–disorder relationship in linker regions is preserved under binary thresholding, confirming that the trend in Figure 3A using continuous mean AIUPred scores is not an artefact of the scoring metric. TMD and NBD disorder fractions remain flat across the full GC range.

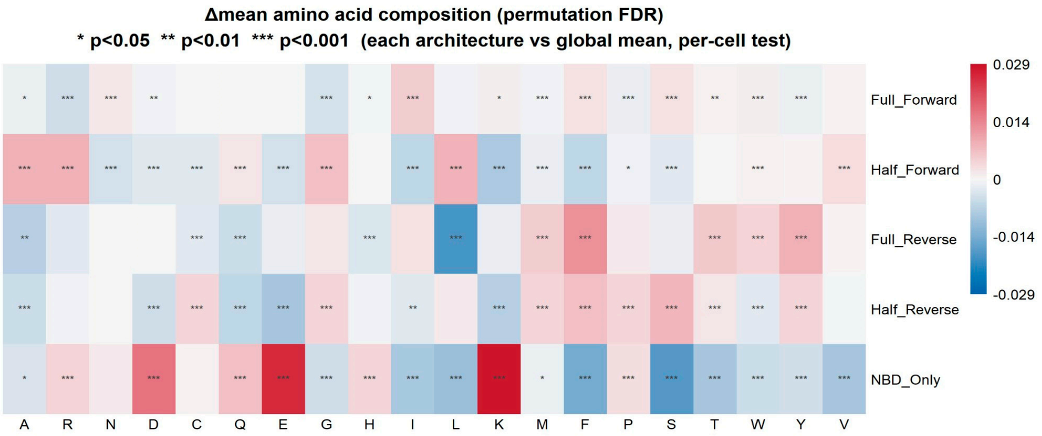

Figure S4. Architecture-specific amino acid composition of ABC transporter linker regions with permutation FDR significance. Heatmap of mean amino acid fraction deviation (Δmean) from the global mean across all five architectural classes (rows) per amino acid (columns, A through V). Red cells = enrichment; blue cells = depletion; colour scale ±0.029. Significance assessed by permutation test (10,000 label shuffles, Benjamini–Hochberg FDR across 100 cells): * $p < 0.05$; ** $p < 0.01$; *** $p < 0.001$. Half_Forward transporters show the broadest significant compositional deviation. NBD_Only proteins show the strongest signals, with glutamate (E) and lysine (K) enriched ($p < 0.001$) and phenylalanine (F) depleted ($p < 0.001$). This figure extends Figure 4K of the main text with full statistical annotation.

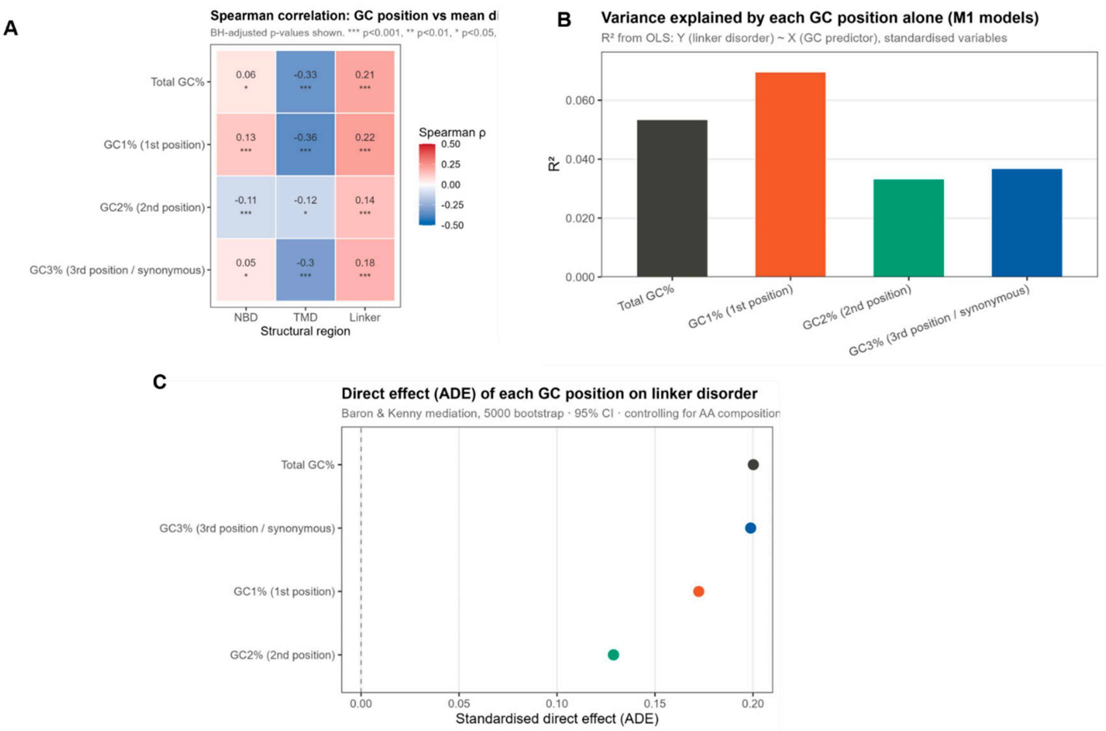

Figure S5. Comparison of GC content at all codon positions versus mean disorder by region type and direct effect on linker disorder. (a) Heatmap of Spearman rank correlations (ϱ) between GC content at each codon position (total GC%, GC1%, GC2%, GC3%; rows) and mean AIUPred disorder score per structural region type (NBD, TMD, linker; columns). BH-adjusted p-values annotated (* $p < 0.05$; ** $p < 0.01$; *** $p < 0.001$). All four GC metrics show positive correlations with linker disorder and negative correlations with TMD disorder. (b) R^2 from OLS regression of mean linker disorder on each GC predictor alone (M1 models, standardised variables, n = 1,581 linker genes); GC2% explains the most variance ($R^2 = 0.164$), confirming the primary role of non-synonymous codon positions. (c) Standardised direct effect (ADE) from Baron and Kenny mediation (5,000 bootstrap resamples) for each GC predictor after controlling for amino acid composition (IDP index as mediator); all four GC metrics retain significant direct effects, with GC2% showing the largest residual effect (ADE = 0.172) and GC3% (synonymous wobble position, ADE = 0.199) comparable to total GC% (ADE = 0.200).

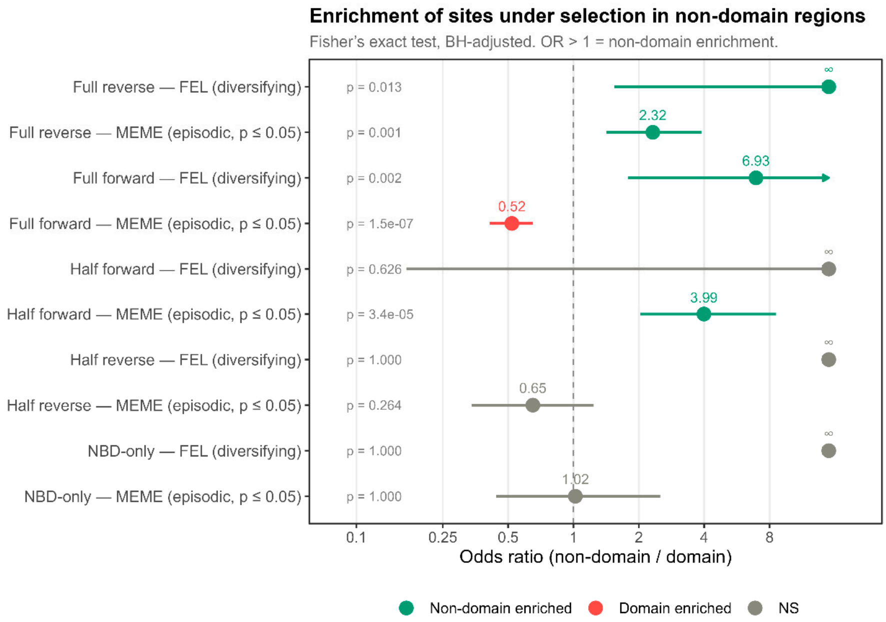

Figure S6. Enrichment of sites under pervasive diversifying selection (FEL) and episodic positive selection (MEME, $p \leq 0.05$) in non-domain versus domain regions across ABC transporter architectural classes. Odds

ratios (OR) with 95% confidence intervals from Fisher's exact tests are shown on a log scale; dashed vertical line indicates OR = 1 (no enrichment). Green = significant non-domain enrichment (BH-adjusted $p < 0.05$); red = significant domain enrichment; grey = not significant. Arrows indicate confidence intervals extending beyond the displayed range. Tests based on fewer than five selected sites (Half_Reverse FEL, NBD-only FEL) are included for completeness but lack statistical power. p-values shown are BH-adjusted across all tests.

Supplementary Tables

Table S1. KEGG organism codes abbreviations for all species represented in the dataset.

Organism codes follow the standard KEGG three- to four-letter abbreviation convention. Kingdom and phylum classifications follow the NCBI taxonomy. Multiple Escherichia coli and Mycobacterium tuberculosis codes correspond to distinct sequenced strains. Scientific names in italics.

KEGG Code	Scientific Name	Common Name	Kingdom	Phylum
**aag**	*Aedes aegypti*	Yellow fever mosquito	Animals	Arthropoda
**apla**	*Anas platyrhynchos*	Mallard	Animals	Chordata
**asn**	*Alligator sinensis*	Chinese alligator	Animals	Chordata
**bta**	*Bos taurus*	Cattle	Animals	Chordata
**cbr**	*Caenorhabditis briggsae*	Nematode	Animals	Nematoda
**cel**	*Caenorhabditis elegans*	Roundworm	Animals	Nematoda
**cmy**	*Chelonia mydas*	Green sea turtle	Animals	Chordata
**dme**	*Drosophila melanogaster*	Fruit fly	Animals	Arthropoda
**dre**	*Danio rerio*	Zebrafish	Animals	Chordata
**ecb**	*Equus caballus*	Horse	Animals	Chordata
**efus**	*Eptesicus fuscus*	Big brown bat	Animals	Chordata
**fca**	*Felis catus*	Domestic cat	Animals	Chordata
**gga**	*Gallus gallus*	Chicken	Animals	Chordata
**hsa**	*Homo sapiens*	Human	Animals	Chordata
**lav**	*Loxodonta africana*	African savanna elephant	Animals	Chordata
**mcc**	*Macaca mulatta*	Rhesus monkey	Animals	Chordata
**mmu**	*Mus musculus*	House mouse	Animals	Chordata

KEGG Code	Scientific Name	Common Name	Kingdom	Phylum
**oaa**	*Ornithorhynchus anatinus*	Platypus	Animals	Chordata
**pcad**	*Physeter macrocephalus*	Sperm whale	Animals	Chordata
**ppad**	*Panthera pardus*	Leopard	Animals	Chordata
**ptet**	*Puntigrus tetrazona*	Sumatra barb	Animals	Chordata
**ptr**	*Pan troglodytes*	Chimpanzee	Animals	Chordata
**rno**	*Rattus norvegicus*	Rat	Animals	Chordata
**tgu**	*Taeniopygia guttata*	Zebra finch	Animals	Chordata
**tru**	*Takifugu rubripes*	Torafugu (tiger pufferfish)	Animals	Chordata
**xla**	*Xenopus laevis*	African clawed frog	Animals	Chordata
**ath**	*Arabidopsis thaliana*	Thale cress	Plants	Streptophyta
**osa**	*Oryza sativa japonica*	Japanese rice	Plants	Streptophyta
**zma**	*Zea mays*	Maize / corn	Plants	Streptophyta
**cal**	*Candida albicans*	Candida	Fungi	Ascomycota
**ena**	*Escherichia coli NA114*	E. coli NA114 (UPEC)	Bacteria	Proteobacteria
**sce**	*Saccharomyces cerevisiae*	Baker's yeast	Fungi	Ascomycota
**ebw**	*Escherichia coli K-12 BW2952*	E. coli K-12 BW2952	Bacteria	Proteobacteria
**ecd**	*Escherichia coli K-12 DH10B*	E. coli K-12 DH10B	Bacteria	Proteobacteria
**ece**	*Escherichia coli O157:H7 EDL933*	E. coli O157:H7 EDL933	Bacteria	Proteobacteria
**ecj**	*Escherichia coli K-12 W3110*	E. coli K-12 W3110	Bacteria	Proteobacteria
**eco**	*Escherichia coli K-12 MG1655*	E. coli K-12 MG1655	Bacteria	Proteobacteria
**ecok**	*Escherichia coli K-12 MDS42*	E. coli K-12 MDS42	Bacteria	Proteobacteria
**ecos**	*Escherichia coli O25b K100 H4-ST131 EC958*	E. coli EC958 (UPEC)	Bacteria	Proteobacteria
**ecs**	*Escherichia coli O157:H7 Sakai*	E. coli O157:H7 Sakai	Bacteria	Proteobacteria

KEGG Code	Scientific Name	Common Name	Kingdom	Phylum
**mra**	*Mycobacterium tuberculosis H37Ra*	M. tuberculosis H37Ra	Bacteria	Actinobacteria
**msm**	*Mycolicibacterium smegmatis MC2 155*	M. smegmatis	Bacteria	Actinobacteria
**mtb**	*Mycobacterium tuberculosis KZN 1435*	M. tuberculosis KZN 1435	Bacteria	Actinobacteria
**mtc**	*Mycobacterium tuberculosis CDC1551*	M. tuberculosis CDC1551	Bacteria	Actinobacteria
**mtf**	*Mycobacterium tuberculosis F11*	M. tuberculosis F11	Bacteria	Actinobacteria
**mtu**	*Mycobacterium tuberculosis H37Rv*	M. tuberculosis H37Rv	Bacteria	Actinobacteria
**mtv**	*Mycobacterium tuberculosis H37Rv*	M. tuberculosis H37Rv (mtv)	Bacteria	Actinobacteria
**pae**	*Pseudomonas aeruginosa PAO1*	P. aeruginosa PAO1	Bacteria	Proteobacteria
**sau**	*Staphylococcus aureus N315*	S. aureus N315 (MRSA)	Bacteria	Firmicutes
**vch**	*Vibrio cholerae O1 El Tor N16961*	Cholera bacterium	Bacteria	Proteobacteria
**hal**	*Halobacterium salinarum NRC-1*	Halobacterium	Archaea	Euryarchaeota
**ddi**	*Dictyostelium discoideum*	Social amoeba	Protists	Amoebozoa
**lma**	*Leishmania major*	Leishmania	Protists	Kinetoplastida
**pfa**	*Plasmodium falciparum 3D7*	Malaria parasite	Protists	Apicomplexa
**tbr**	*Trypanosoma brucei brucei*	Trypanosome	Protists	Kinetoplastida
**tgo**	*Toxoplasma gondii*	Toxoplasma	Protists	Apicomplexa

Note. *KEGG: Kyoto Encyclopedia of Genes and Genomes (https://www.genome.jp). 'Animals' includes all Metazoa. 'Protists' include protozoan eukaryotes (Apicomplexa, Kinetoplastida, Ciliophora, Amoebozoa).*

Table S2. Dataset composition per architectural class.

Summary of sequence counts and taxonomic coverage per architectural class after duplicate removal and quality filtering. 'Yes' = at least one sequence from that taxonomic group; '—' = absent or not applicable. Seven additional sequences corresponding to prokaryotic ABC transporters not conforming to eukaryotic architectural class definitions were retained

in superfamily-wide regression analyses (n = 1,581) but excluded from class-stratified analyses.

Architectural class	n	Subfamilies	Prokaryotes	Fungi	Plants	Invertebrates	Mammals	Birds	Reptiles/Fish/ Amphibians	Protists
Full forward (TMD–NBD–TMD–NBD)	751	ABCA, ABCB, ABCC	Yes	Yes	Yes	Yes	Yes	Yes	Yes	Yes
Full reverse (NBD–TMD–NBD–TMD; full-size ABCG/PDR)	69	ABCG	—	Yes	Yes	—	—	—	—	Yes
Half forward (TMD–NBD)	372	ABCA, ABCB, ABCC, ABCD	Yes	Yes	Yes	Yes	Yes	Yes	Yes	Yes
Half reverse (NBD–TMD)	228	ABCG	Yes	Yes	Yes	Yes	Yes	Yes	Yes	Yes
NBD-only (NBD–NBD)	154	ABCE, ABCF	Yes	Yes	Yes	Yes	Yes	Yes	Yes	Yes
**Total (5 classes)**	**1,574**	**ABCA–ABCG**	—	—	—	—	—	—	—	—

Note. *The full reverse class comprises full-size ABCG transporters of the pleiotropic drug resistance (PDR) type, restricted to plants, fungi, and protists. Minor subfamily contributions in the half forward class include 2 ABCA and 23 ABCC sequences. One ABCC sequence initially classified as full reverse was reclassified after manual inspection. 'Invertebrates' includes insects and nematodes. 'Reptiles/Fish/Amphibians' includes all non-avian, non-mammalian jawed vertebrates and amphibians. Seven additional sequences correspond to prokaryotic-type ABC transporters that do not conform to the five eukaryotic architectural class definitions; these were included as outgroup sequences during phylogenetic tree inference and were assigned to the nearest architectural class on the basis of sequence similarity, where they are labelled "Other" Including these seven sequences, the total dataset comprises 1,581 sequences.*

Table S3. Comparison of disorder metrics and protein length between full reverse and half reverse ABC transporters.

Mann–Whitney U tests with Cliff's delta effect sizes. Both classes comprise ABCG subfamily members but differ in domain architecture (full reverse: NBD–TMD–NBD–TMD, full-size ABCG/PDR; half reverse: NBD–TMD, canonical ABCG half-transporter). Values are medians. Sorted by statistical significance.

Metric	Full reverse (n = 69)	Half reverse (n = 228)	p-value	Cliff's δ	Magnitude
**Linker disorder fraction**	0.385	0.071	**9.51×10^{-23}**	**0.775**	*large*
**Protein length (aa)**	1,449	663	**2.77×10^{-36}**	**0.999**	*large*
**Cflank disorder fraction**	0.355	0.227	**0.008**	**0.209**	*small*
Overall disorder	0.089	0.078	0.198	0.102	*negligible*

fraction					
Nflank disorder fraction	0.288	0.288	0.344	0.075	*negligible*

Note. *Significant comparisons (p < 0.05) shown in bold with red p-values. Cliff's δ magnitude thresholds: negligible (|δ| < 0.147), small (0.147–0.33), medium (0.33–0.474), large (|δ| > 0.474), per Romano et al. (2006).*

Table S4. Cross-architecture pairwise comparisons of regional intrinsic disorder fraction and linker length.

Mann-Whitney U tests (two-sided) with Benjamini-Hochberg correction within each comparison family. Cliff's d thresholds: large |d| ≥ 0.474, medium ≥ 0.330, small ≥ 0.147, negligible < 0.147.

Group 1	Group 2	n_1	n_2	Median 1	Median 2	p (BH-adj)	Sig.	Cliff's d	Effect
**Cflank disorder**									
Full_Forward	Half_Reverse	759	228	0.000	0.227	< 0.001	***	-0.787	large
Half_Reverse	NBD_Only	228	153	0.227	0.000	< 0.001	***	0.795	large
Full_Forward	Full_Reverse/ABCG	759	70	0.000	0.353	< 0.001	***	-0.827	large
Full_Reverse/ABCG	NBD_Only	70	153	0.353	0.000	< 0.001	***	0.826	large
Half_Forward	Half_Reverse	369	228	0.029	0.227	< 0.001	***	-0.475	large
Full_Forward	Half_Forward	759	369	0.000	0.029	< 0.001	***	-0.279	small
Full_Reverse/ABCG	Half_Forward	70	369	0.353	0.029	< 0.001	***	0.596	large
Half_Forward	NBD_Only	369	153	0.029	0.000	< 0.001	***	0.279	small
Full_Reverse/ABCG	Half_Reverse	70	228	0.353	0.227	0.016	*	0.192	small
Full_Forward	NBD_Only	759	153	0.000	0.000	0.666	ns	0.018	negligible
**Linker disorder**									
Half_Forward	Half_Reverse	366	228	0.000	0.071	< 0.001	***	-0.470	medium
Full_Forward	Full_Reverse/ABCG	752	68	0.380	0.081	< 0.001	***	0.597	large

Group 1	Group 2	n_1	n_2	Median 1	Median 2	p (BH-adj)	Sig.	Cliff's d	Effect
Full_Forward	Half_Forward	752	366	0.380	0.000	< 0.001	***	0.804	large
Full_Forward	Half_Reverse	752	228	0.380	0.071	< 0.001	***	0.576	large
Full_Reverse/ABCG	Half_Forward	68	366	0.081	0.000	< 0.001	***	0.706	large
Full_Reverse/ABCG	Half_Reverse	68	228	0.081	0.071	0.327	ns	0.078	negligible
**Linker length**									
Half_Forward	Half_Reverse	366	228	69	155	< 0.001	***	-0.944	large
Full_Forward	Full_Reverse/ABCG	752	68	177	817.5	< 0.001	***	-1.000	large
Full_Forward	Half_Forward	752	366	177	69	< 0.001	***	0.970	large
Full_Forward	Half_Reverse	752	228	177	155	< 0.001	***	0.470	medium
**Nflank disorder**									
Full_Forward	Full_Reverse/ABCG	757	70	0.178	0.285	0.212	ns	-0.144	negligible
Full_Forward	NBD_Only	757	100	0.178	0.383	0.212	ns	-0.099	negligible

Note. *N-terminal flank disorder did not differ significantly across any architectural class pair (all BH-adjusted $p > 0.05$; Cliff's d −0.148 to +0.128). Comparison families: (i) primary linker disorder fraction; (ii) primary linker length; (iii) C-terminal flank disorder; (iv) N-terminal flank disorder.*

Table S5. Tiered predicted PTM conservation sites across ABC transporter architectural classes.

Predicted PTM sites satisfying a tiered conservation criterion based on MusiteDeep score and cross-species conservancy. High confidence: score ≥ 0.5 AND conservancy ≥ 70% (n = 7 sites). Moderate: score ≥ 0.5 AND conservancy 50–69% (n = 33 sites). Candidate: score ≥ 0.5 AND conservancy 30–49% (n = 100 sites). Total: 140 sites across all architectures. Alignment pos. = position in the trimmed multiple sequence alignment. n passing = number of sequences with MusiteDeep score ≥ 0.5 at this position. n with residue = sequences carrying the correct chemical residue at this position. No. species = number of distinct species contributing to n passing. Species = KEGG organism codes of species with a passing prediction at this site (see Table S1 for organism code abbreviations).

Architecture	Alignment pos.	PTM type	Tier	Conservancy (%)	Max. *score*	n *passing*	n *with residue*	No. species	Species
Full_Reverse	1129	Phosphoserine	Moderate	60.0%	0.784	3	5	2	osa, zma
	91	Phosphoserine	Moderate	60.0%	0.671	3	5	2	osa, zma
	441	Ubiquitination	Moderate	53.6%	0.664	30	56	3	ath, osa, zma

Architecture	Alignment pos.	PTM type	Tier	Conservancy (%)	Max. *score*	n *passing*	n *with residue*	No. species	Species
	78	Phosphoserine	Moderate	50.0%	0.857	3	6	2	osa, zma
	53	Phosphoserine	Moderate	50.0%	0.822	7	14	2	osa, zma
	93	Phosphoserine	Moderate	50.0%	0.795	8	16	4	ath, ddi, osa, zma
	961	N-linked glycosylation	Candidate	46.7%	0.917	7	15	3	cal, ddi, sce
	41	Phosphoserine	Candidate	45.7%	0.856	21	46	4	ath, ddi, osa, zma
	61	Phosphoserine	Candidate	43.5%	0.874	20	46	4	ath, ddi, osa, zma
	82	Phosphoserine	Candidate	42.9%	0.779	6	14	2	ath, osa
	1435	Phosphotyrosine	Candidate	40.8%	0.856	29	71	4	ath, ddi, osa, zma
	47	Phosphoserine	Candidate	40.0%	0.904	6	15	2	osa, zma
	1624	Phosphoserine	Candidate	37.1%	0.876	26	70	3	ath, osa, zma
	45	Phosphoserine	Candidate	37.1%	0.860	13	35	3	ath, osa, zma
	69	Phosphoserine	Candidate	36.4%	0.902	4	11	3	ath, osa, zma
	1660	S-palmitoyl cysteine	Candidate	34.3%	0.891	24	70	3	ath, osa, zma
	59	Phosphoserine	Candidate	34.0%	0.799	16	47	4	ath, cal, osa, zma
	96	Phosphoserine	Candidate	33.9%	0.832	19	56	3	ath, osa, zma
	60	Phosphoserine	Candidate	32.6%	0.857	15	46	3	ath, osa, zma
	1053	Pyrrolidone carboxylic acid	Candidate	32.4%	0.907	22	68	3	ath, osa, zma
	1429	Methylarginine	Candidate	32.4%	0.814	23	71	4	ath, osa, sce, zma
	98	Phosphoserine	Candidate	32.1%	0.901	18	56	5	ath, ddi, osa, sce, zma
	431	SUMOylation	Candidate	32.1%	0.860	17	53	3	ath, osa, zma
	81	Phosphoserine	Candidate	31.1%	0.728	14	45	3	ath, osa, zma
	1726	N-linked glycosylation	Candidate	31.0%	0.889	22	71	3	ath, osa, zma
	1000	N6-acetyllysine	Candidate	30.8%	0.772	4	13	1	sce
	97	Phosphoserine	Candidate	30.4%	0.853	17	56	5	ath, cal, osa, sce, zma
Half_Forward	203	S-palmitoyl cysteine	Moderate	59.5%	0.860	25	42	21	apla, bta, cmy, dme, dre, ecb, efus, fca, gga, hsa, lav, mcc, mmu, pcad, ppad, ptet, ptr, rno, tgu, tru, xla
	189	Hydroxyproline	Moderate	56.2%	0.859	9	16	9	ecb, fca, hsa, lav, mcc, oaa, pcad, ppad, ptr
	1914	SUMOylation	Moderate	52.0%	0.862	13	25	11	dre, efus, hsa, mcc, mmu, oaa, ptet, ptr, rno, tru, xla
	1129	N-linked glycosylation	Moderate	51.4%	0.910	19	37	19	apla, bta, cmy, dre, ecb, efus, fca, gga, hsa, lav, mcc, mmu, oaa, pcad, ppad, ptet, ptr, rno, xla
	2938	S-palmitoyl cysteine	Moderate	51.1%	0.913	23	45	23	apla, asn, bta, cbr, cmy, dre, ecb, efus, fca, gga, hsa, lav, mcc, mmu, oaa, pcad, ppad, ptet, ptr, rno, tgu, tru, xla
	1908	Ubiquitination	Candidate	42.2%	0.669	19	45	16	apla, asn, bta, cmy, ecb, efus, fca, hsa, lav, mcc, mmu, oaa, pcad, ppad, ptr, rno
	2943	S-palmitoyl cysteine	Candidate	40.4%	0.702	23	57	23	apla, asn, bta, cbr, cmy, dre, ecb, efus, fca, gga, hsa, lav, mcc, mmu, oaa, pcad, ppad, ptet, ptr, rno,

Architecture	Alignment pos.	PTM type	Tier	Conservancy (%)	Max. *score*	n *passing*	n *with residue*	No. species	Species
									tgu, tru, xla
	2763	Pyrrolidone carboxylic acid	Candidate	38.8%	0.680	19	49	16	apla, asn, cmy, efus, fca, gga, hsa, lav, mcc, mmu, oaa, pcad, ptr, rno, tgu, xla
	687	Pyrrolidone carboxylic acid	Candidate	38.5%	0.518	5	13	5	hsa, mcc, mmu, ptr, rno
	2591	Ubiquitination	Candidate	37.6%	0.734	138	367	36	aag, apla, asn, ath, bta, cbr, cmy, ddi, dme, dre, ebw, ecb, ece, ecos, ecs, efus, fca, gga, hsa, lav, mcc, mmu, msm, oaa, osa, pcad, ppad, ptet, ptr, rno, sce, tbr, tgu, tru, xla, zma
	101	Phosphoserine	Candidate	37.5%	0.823	3	8	3	cmy, mcc, ptr
	151	Hydroxyproline	Candidate	37.5%	0.806	3	8	3	cmy, efus, ptr
	2940	N-linked glycosylation	Candidate	33.8%	0.879	22	65	22	apla, asn, bta, cmy, dre, ecb, efus, fca, gga, hsa, lav, mcc, mmu, oaa, pcad, ppad, ptet, ptr, rno, tgu, tru, xla
	87	Methylarginine	Candidate	33.3%	0.921	3	9	3	cmy, mcc, ptr
	190	Methylarginine	Candidate	33.3%	0.863	5	15	5	bta, hsa, mcc, oaa, ptr
	688	N6-acetyllysine	Candidate	30.8%	0.658	4	13	4	bta, ecb, efus, pcad
	2879	O-linked glycosylation	Candidate	30.0%	0.630	3	10	3	hsa, mcc, ptr
Half_Reverse	1814	Phosphoserine	**High confidence**	75.0%	0.888	9	12	3	ath, osa, zma
	1928	Phosphoserine	Moderate	63.6%	0.904	7	11	3	ath, osa, zma
	856	Phosphoserine	Moderate	57.5%	0.906	111	193	25	apla, asn, ath, bta, ddi, dre, ecb, efus, fca, gga, hsa, lav, mcc, mmu, oaa, osa, pcad, ppad, ptet, ptr, rno, tgu, tru, xla, zma
	1812	Phosphoserine	Moderate	53.8%	0.864	7	13	3	ath, osa, zma
	303	N-linked glycosylation	Moderate	50.0%	0.889	13	26	13	apla, asn, bta, ecb, efus, fca, gga, hsa, lav, mcc, mmu, ppad, rno
	25	S-palmitoyl cysteine	Moderate	50.0%	0.781	18	36	17	apla, bta, dre, ecb, efus, fca, gga, hsa, lav, mcc, mmu, oaa, ppad, ptet, rno, tgu, xla
	288	S-palmitoyl cysteine	Candidate	43.8%	0.650	7	16	7	bta, ecb, fca, hsa, mmu, pcad, rno
	1083	Ubiquitination	Candidate	42.9%	0.695	3	7	3	apla, gga, tgu
	845	Methylarginine	Candidate	39.2%	0.857	56	143	26	apla, asn, ath, bta, cmy, ddi, dre, ecb, efus, fca, hsa, lav, mcc, mmu, oaa, osa, pcad, ppad, ptet, ptr, rno, tgo, tgu, tru, xla, zma
	1799	Phosphoserine	Candidate	38.5%	0.830	5	13	3	ath, osa, zma
	319	Phosphoserine	Candidate	37.5%	0.823	3	8	3	dre, ptet, zma
	1576	Methylarginine	Candidate	35.7%	0.687	5	14	2	osa, zma
	265	Phosphoserine	Candidate	33.3%	0.894	6	18	5	ath, osa, ptet, tgo, zma
	160	Phosphoserine	Candidate	33.3%	0.858	3	9	2	ath, zma
	1637	N-linked	Candidate	32.4%	0.926	23	71	19	bta, cmy, dre, ecb,

Architecture	Alignment pos.	PTM type	Tier	Conservancy (%)	Max. *score*	n *passing*	n *with residue*	No. species	Species
		glycosylation							efus, fca, hsa, lav, mcc, mmu, oaa, pcad, ppad, ptet, ptr, rno, tgu, tru, xla
	1643	N-linked glycosylation	Candidate	31.8%	0.922	14	44	13	asn, bta, cmy, ecb, efus, fca, lav, mcc, mmu, oaa, pcad, ppad, rno
	1631	N-linked glycosylation	Candidate	31.7%	0.922	40	126	22	apla, asn, bta, dre, ecb, efus, fca, gga, hsa, lav, mcc, mmu, oaa, osa, pcad, ppad, ptet, ptr, rno, tgu, tru, xla
NBD_Only	563	Phosphoserine	**High confidence**	80.0%	0.840	4	5	3	dre, ptet, tru
	1644	Phosphotyrosine	**High confidence**	78.6%	0.906	33	42	29	aag, apla, asn, ath, bta, cal, cmy, ddi, dre, ecb, efus, fca, gga, hal, hsa, lav, lma, oaa, osa, pcad, pfa, ptet, rno, sce, tgo, tgu, tru, xla, zma
	110	Methyllysine	**High confidence**	77.8%	0.743	14	18	14	aag, bta, cmy, ecb, efus, fca, hsa, lav, mcc, mmu, pcad, ppad, ptr, rno
	612	Phosphothreonine	**High confidence**	73.3%	0.649	11	15	11	bta, ecb, efus, fca, gga, lav, mcc, mmu, ppad, rno, tgu
	1629	Phosphoserine	Moderate	66.7%	0.784	34	51	31	aag, apla, asn, ath, bta, cal, cbr, cmy, ddi, dme, dre, ecb, efus, fca, gga, hal, hsa, lav, lma, oaa, osa, pcad, pfa, ptet, rno, sce, tbr, tgu, tru, xla, zma
	318	Phosphoserine	Moderate	65.5%	0.928	19	29	18	asn, bta, cmy, dre, ecb, efus, fca, hsa, lav, mcc, mmu, pcad, ppad, ptet, ptr, rno, tru, xla
	1115	Phosphoserine	Moderate	63.0%	0.891	17	27	16	asn, bta, dre, ecb, efus, fca, hsa, lav, mcc, mmu, pcad, ppad, ptet, ptr, rno, tru
	548	Phosphoserine	Moderate	61.1%	0.927	11	18	11	bta, ecb, efus, fca, hsa, lav, mmu, pcad, ppad, rno, xla
	1641	Phosphoserine	Moderate	57.8%	0.720	26	45	23	apla, asn, bta, cal, cbr, cmy, dme, dre, ecb, efus, fca, gga, hsa, lav, lma, oaa, pcad, ptet, rno, sce, tgu, tru, xla
	392	Phosphoserine	Moderate	57.1%	0.922	4	7	4	aag, dme, dre, ptet
	858	Phosphoserine	Moderate	50.0%	0.865	3	6	3	cal, sce, zma
	582	Phosphotyrosine	Moderate	50.0%	0.836	12	24	12	asn, bta, cmy, ecb, efus, fca, hsa, lav, mmu, pcad, ppad, rno
	492	Ubiquitination	Candidate	47.6%	0.590	10	21	10	asn, bta, cmy, ecb, efus, fca, lav, mcc, pcad, ppad
	294	Methylarginine	Candidate	45.5%	0.789	10	22	10	bta, ecb, efus, fca, hsa, lav, mcc, pcad, ppad, ptr

Architecture	Alignment pos.	PTM type	Tier	Conservancy (%)	Max. *score*	n *passing*	n *with residue*	No. species	Species
	771	N6-acetyllysine	Candidate	43.7%	0.689	66	151	31	aag, apla, asn, ath, bta, cal, cbr, cmy, ddi, dme, dre, ecb, efus, fca, gga, hsa, lav, mcc, mmu, oaa, pcad, pfa, ppad, ptet, ptr, rno, sce, tgo, tgu, tru, xla
	132	Phosphoserine	Candidate	41.4%	0.894	12	29	12	bta, ecb, efus, fca, hsa, lav, mcc, mmu, pcad, ppad, ptr, rno
	268	Phosphoserine	Candidate	41.3%	0.927	19	46	18	asn, bta, cmy, dre, ecb, efus, fca, hsa, lav, mcc, mmu, pcad, ppad, ptet, ptr, rno, tru, xla
	921	Phosphothreonine	Candidate	40.0%	0.859	46	115	27	apla, asn, ath, bta, cbr, cmy, dre, ecb, efus, fca, gga, hsa, lav, mcc, mmu, oaa, osa, pcad, ppad, ptet, ptr, rno, tbr, tgu, tru, xla, zma
	542	SUMOylation	Candidate	38.9%	0.762	7	18	7	bta, ecb, efus, fca, lav, pcad, ppad
	108	Hydroxyproline	Candidate	38.3%	0.766	41	107	26	aag, asn, bta, cbr, cmy, dre, ecb, efus, fca, gga, hsa, lav, lma, mcc, mmu, oaa, pcad, ppad, ptet, ptr, rno, sce, tbr, tgu, tru, xla
	129	Phosphothreonine	Candidate	37.0%	0.600	10	27	10	bta, ecb, fca, hsa, lav, mcc, mmu, ppad, ptr, rno
	906	Ubiquitination	Candidate	36.9%	0.699	41	111	23	apla, asn, bta, cbr, cmy, dre, ecb, efus, fca, gga, hsa, lav, mcc, mmu, oaa, pcad, ppad, ptet, ptr, rno, tgu, tru, xla
	254	Phosphoserine	Candidate	36.4%	0.896	20	55	19	asn, bta, cmy, dme, dre, ecb, efus, fca, hsa, lav, mcc, mmu, pcad, ppad, ptet, ptr, rno, tru, xla
	113	Methyllysine	Candidate	35.0%	0.741	7	20	7	dme, dre, hsa, mcc, ptet, ptr, tru
	498	Phosphoserine	Candidate	33.3%	0.929	7	21	6	bta, cmy, lav, mmu, rno, tru
	452	N6-acetyllysine	Candidate	33.3%	0.760	44	132	29	aag, apla, asn, ath, bta, cal, cbr, cmy, dme, dre, ecb, efus, fca, gga, hsa, lav, lma, mcc, mmu, oaa, osa, ppad, ptet, rno, sce, tgu, tru, xla, zma
	677	Phosphoserine	Candidate	32.3%	0.830	21	65	20	aag, asn, bta, cmy, dme, dre, ecb, efus, fca, hsa, lav, mcc, mmu, pcad, ppad, ptet, ptr, rno, tru, xla
	240	Phosphoserine	Candidate	30.9%	0.907	30	97	24	aag, ath, bta, cbr, cmy, dme, dre, ecb, efus, fca, gga, hsa, lav, mcc, mmu, oaa, osa, ppad, ptet, rno, tgu, tru, xla, zma

Architecture	Alignment pos.	PTM type	Tier	Conservancy (%)	Max. *score*	n *passing*	n *with residue*	No. species	Species
	1030	N6-acetyllysine	Candidate	30.6%	0.816	34	111	27	aag, apla, asn, ath, bta, cmy, ddi, dme, dre, ecb, efus, fca, gga, hsa, lav, mcc, mmu, oaa, osa, pcad, ppad, ptet, rno, tgu, tru, xla, zma
	712	N-linked glycosylation	Candidate	30.2%	0.906	42	139	22	apla, asn, bta, cmy, dre, ecb, efus, fca, gga, hsa, lav, mcc, mmu, oaa, pcad, ppad, ptet, ptr, rno, tgu, tru, xla
	880	Ubiquitination	Candidate	30.0%	0.552	3	10	3	cal, osa, sce
Full_Forward	5987	Phosphoserine	**High confidence**	71.4%	0.876	15	21	14	apla, bta, cmy, ecb, efus, fca, gga, hsa, oaa, ppad, ptr, rno, tgu, xla
	5522	Phosphothreonine	**High confidence**	70.0%	0.861	14	20	13	apla, asn, cmy, ecb, efus, fca, gga, hsa, oaa, pcad, rno, tgu, xla
	4982	N-linked glycosylation	Moderate	60.0%	0.930	18	30	16	apla, asn, bta, dre, ecb, efus, fca, gga, hsa, oaa, pcad, ppad, ptet, rno, tgu, xla
	5518	Phosphoserine	Moderate	58.3%	0.895	14	24	13	apla, asn, cmy, ecb, efus, fca, gga, hsa, oaa, pcad, rno, tgu, xla
	421	N-linked glycosylation	Moderate	56.2%	0.901	9	16	9	bta, ecb, efus, fca, hsa, lav, pcad, ppad, ptr
	5942	Phosphoserine	Moderate	55.6%	0.909	15	27	14	apla, bta, cmy, dme, ecb, efus, fca, gga, hsa, oaa, ppad, ptr, tgu, xla
	100	N-linked glycosylation	Moderate	55.1%	0.922	81	147	20	aag, apla, asn, bta, cmy, dre, ecb, efus, fca, gga, hsa, oaa, pcad, ppad, ptet, ptr, rno, tgu, tru, xla
	448	N-linked glycosylation	Moderate	52.9%	0.908	9	17	9	bta, ecb, efus, fca, hsa, lav, pcad, ppad, ptr
	729	N-linked glycosylation	Moderate	52.6%	0.919	10	19	10	bta, cmy, ecb, efus, fca, hsa, mmu, ppad, ptr, rno
	500	N-linked glycosylation	Moderate	52.4%	0.911	11	21	11	asn, bta, ecb, efus, fca, hsa, lav, pcad, ppad, ptr, tgu
	621	N-linked glycosylation	Moderate	50.0%	0.919	10	20	10	apla, cmy, ecb, fca, gga, hsa, mmu, ppad, ptr, rno
	6133	Phosphoserine	Candidate	47.8%	0.783	11	23	11	bta, cmy, ecb, efus, fca, gga, hsa, oaa, ppad, ptr, rno
	780	Phosphoserine	Candidate	47.4%	0.890	9	19	9	bta, ecb, efus, fca, hsa, mmu, ppad, ptr, rno
	4507	O-linked glycosylation	Candidate	47.4%	0.777	9	19	9	dre, ecb, efus, fca, hsa, pcad, ppad, ptet, rno
	335	Phosphoserine	Candidate	45.5%	0.848	10	22	10	apla, bta, ecb, efus, fca, hsa, lav, pcad, ppad, ptr
	308	Phosphoserine	Candidate	45.0%	0.805	9	20	9	bta, ecb, efus, fca, hsa, lav, pcad, ppad, ptr
	5866	Phosphoserine	Candidate	43.5%	0.864	10	23	10	apla, cmy, ecb, fca, gga, hsa, oaa, ppad, ptr, tgu
	5982	Phosphoserine	Candidate	43.3%	0.771	13	30	12	bta, ddi, ecb, efus, fca, hsa, oaa, pcad, ppad,

Architecture	Alignment pos.	PTM type	Tier	Conservancy (%)	Max. *score*	n *passing*	n *with residue*	No. species	Species
									ptr, rno, xla
	5975	Phosphoserine	Candidate	42.9%	0.902	12	28	11	asn, bta, ecb, efus, fca, hsa, oaa, ppad, ptr, rno, xla
	264	N-linked glycosylation	Candidate	38.5%	0.905	15	39	15	apla, asn, bta, cmy, ecb, efus, fca, gga, hsa, mmu, ppad, ptr, rno, tru, xla
	3999	N-linked glycosylation	Candidate	38.2%	0.918	13	34	9	bta, ecb, efus, fca, hsa, lav, pcad, ppad, ptr
	4029	N-linked glycosylation	Candidate	38.1%	0.905	8	21	8	bta, cmy, ecb, efus, hsa, lav, pcad, ptr
	298	Phosphoserine	Candidate	38.1%	0.709	8	21	8	bta, ecb, efus, fca, hsa, pcad, ppad, ptr
	2861	N-linked glycosylation	Candidate	37.5%	0.905	6	16	6	cmy, ecb, fca, hsa, mmu, ptr
	1380	N-linked glycosylation	Candidate	36.8%	0.905	7	19	7	apla, ecb, fca, hsa, mmu, ppad, ptr
	4506	O-linked glycosylation	Candidate	36.8%	0.809	7	19	6	dre, efus, fca, pcad, ptet, tru
	3568	N-linked glycosylation	Candidate	35.7%	0.920	5	14	5	ecb, fca, hsa, ppad, ptr
	4505	N-linked glycosylation	Candidate	35.0%	0.919	7	20	7	dre, efus, fca, hsa, pcad, ptet, rno
	2708	N-linked glycosylation	Candidate	35.0%	0.910	7	20	7	ecb, fca, hsa, mmu, ppad, ptr, tru
	4986	N-linked glycosylation	Candidate	33.3%	0.924	8	24	8	apla, asn, cmy, efus, fca, gga, hsa, ptet
	1927	N-linked glycosylation	Candidate	33.3%	0.919	8	24	8	apla, fca, gga, hsa, mmu, ppad, ptr, xla
	2059	N-linked glycosylation	Candidate	33.3%	0.917	6	18	6	ecb, fca, hsa, mmu, ppad, ptr
	1919	N-linked glycosylation	Candidate	33.3%	0.908	8	24	8	apla, cmy, ecb, fca, hsa, mmu, ppad, ptr
	517	N-linked glycosylation	Candidate	32.6%	0.907	30	92	17	apla, bta, cmy, ecb, efus, fca, gga, hsa, lav, mmu, pcad, ppad, ptet, ptr, rno, tru, xla
	1156	Phosphoserine	Candidate	31.8%	0.863	7	22	7	cmy, ecb, fca, hsa, mmu, ppad, ptr
	4508	O-linked glycosylation	Candidate	31.8%	0.808	7	22	7	apla, asn, dre, ecb, gga, pcad, ppad
	2465	N-linked glycosylation	Candidate	31.6%	0.921	6	19	6	ecb, fca, hsa, mmu, ppad, ptr
	1530	N-linked glycosylation	Candidate	31.2%	0.919	5	16	5	ecb, fca, mmu, ppad, xla
	3581	N-linked glycosylation	Candidate	31.2%	0.914	5	16	5	ecb, fca, hsa, ppad, ptr
	3797	N-linked glycosylation	Candidate	31.2%	0.905	5	16	5	ecb, fca, hsa, ppad, ptr
	3475	N-linked glycosylation	Candidate	31.2%	0.904	5	16	5	ecb, fca, hsa, ppad, ptr
	3544	Phosphoserine	Candidate	31.2%	0.866	5	16	5	ecb, fca, hsa, ppad, ptr
	4052	N-linked glycosylation	Candidate	30.6%	0.907	11	36	10	bta, ecb, efus, fca, hsa, lav, pcad, ppad, ptr, rno
	5660	Phosphoserine	Candidate	30.6%	0.880	15	49	13	asn, bta, ecb, efus, fca, gga, hsa, pcad, ppad, ptet, ptr, rno, tgu
	892	N-linked glycosylation	Candidate	30.0%	0.909	6	20	6	bta, ecb, hsa, mmu, ptr, rno
	309	N-linked glycosylation	Candidate	30.0%	0.882	6	20	6	bta, efus, fca, lav, pcad, ppad

Architecture	Alignment pos.	PTM type	Tier	Conservancy (%)	Max. *score*	n *passing*	n *with residue*	No. species	Species
	6171	Phosphoserine	Candidate	30.0%	0.878	6	20	4	asn, ath, osa, zma
	5992	Phosphothreonine	Candidate	30.0%	0.862	6	20	6	ecb, efus, fca, hsa, ppad, ptr

Note. High-confidence sites (conservancy ≥ 70%) shown in bold. KEGG organism codes follow standard three- to four-letter abbreviations (see Table S1 for full species names). Architecture labels consistent with main text.

Table S6. Within-architecture pairwise comparisons of region length and disorder fraction.

Kruskal–Wallis test with BH-corrected pairwise Wilcoxon comparisons within each architectural class. KW p-value = overall Kruskal–Wallis p-value for that architecture and metric. **** $p < 0.0001$, *** $p < 0.001$, ** $p < 0.01$, * $p < 0.05$, ns = not significant. Region counts (n_1, n_2) reflect the number of sequences with a definable region (≥10 residues) for each comparison and may be slightly less than the total class size. Half_Forward = TMD–NBD; Half_Reverse = NBD–TMD.

Architecture	Metric	KW p-value	Group 1	Group 2	n_1	n_2	p (BH-adj)	Sig.
Full_Forward	length	< 0.001	Nflank	L1	749	751	< 0.001	****
			Nflank	L2	749	751	< 0.001	****
			Nflank	L3	749	751	< 0.001	****
			Nflank	Cflank	749	750	< 0.001	****
			L1	L2	751	751	< 0.001	****
			L1	L3	751	751	< 0.001	****
			L1	Cflank	751	750	< 0.001	****
			L2	L3	751	751	< 0.001	****
			L2	Cflank	751	750	< 0.001	****
			L3	Cflank	751	750	< 0.001	****
Full_Reverse	length	< 0.001	Nflank	L1	69	69	< 0.001	****
			Nflank	L2	69	69	< 0.001	****
			Nflank	L3	69	69	< 0.001	****
			Nflank	Cflank	69	69	< 0.001	****
			L1	L2	69	69	< 0.001	***
			L1	L3	69	69	< 0.001	****
			L1	Cflank	69	69	< 0.001	****
			L2	L3	69	69	0.818	ns
			L2	Cflank	69	69	< 0.001	****
			L3	Cflank	69	69	< 0.001	****
Half_Forward	length	< 0.001	Nflank	L1	368	372	< 0.001	****
			Nflank	Cflank	368	372	< 0.001	****
			L1	Cflank	372	372	0.193	ns
Half_Reverse	length	< 0.001	Nflank	L1	227	228	< 0.001	****
			Nflank	Cflank	227	228	< 0.001	****
			L1	Cflank	228	228	< 0.001	****
NBD_only	length	< 0.001	Nflank	Cflank	154	154	< 0.001	****
Full_Forward	disorder_frac	< 0.001	Nflank	L1	749	751	< 0.001	****
			Nflank	L2	749	751	< 0.001	****
			Nflank	L3	749	751	< 0.001	****
			Nflank	Cflank	749	750	< 0.001	****
			L1	L2	751	751	< 0.001	****
			L1	L3	751	751	0.386	ns
			L1	Cflank	751	750	< 0.001	****
			L2	L3	751	751	< 0.001	****
			L2	Cflank	751	750	< 0.001	****
			L3	Cflank	751	750	< 0.001	****
Full_Reverse	disorder_frac	< 0.001	Nflank	L1	69	69	< 0.001	****

Architecture	Metric	KW p-value	Group 1	Group 2	n_1	n_2	p (BH-adj)	Sig.
			Nflank	L2	69	69	0.008	**
			Nflank	L3	69	69	< 0.001	****
			Nflank	Cflank	69	44	0.168	ns
			L1	L2	69	69	< 0.001	****
			L1	L3	69	69	0.297	ns
			L1	Cflank	69	44	< 0.001	****
			L2	L3	69	69	< 0.001	****
			L2	Cflank	69	44	< 0.001	***
			L3	Cflank	69	44	< 0.001	****
Half_Forward	disorder_frac	< 0.001	Nflank	L1	368	372	< 0.001	****
			Nflank	Cflank	368	372	< 0.001	****
			L1	Cflank	372	372	< 0.001	****
Half_Reverse	disorder_frac	< 0.001	Nflank	L1	227	228	< 0.001	****
			Nflank	Cflank	227	228	0.033	*
			L1	Cflank	228	228	< 0.001	****
NBD_only	disorder_frac	< 0.001	Nflank	Cflank	154	154	< 0.001	****

Note. *Region abbreviations: Nflank = N-terminal flanking region; Cflank = C-terminal flanking region; L1, L2, L3 = inter-domain linkers (full transporters); L1 = single inter-domain linker (half transporters). Architecture abbreviations: Full_Forward = full forward (TMD–NBD–TMD–NBD); Full_Reverse = full reverse (NBD–TMD–NBD–TMD; full-size ABCG/PDR); Half_Forward = half forward (TMD–NBD); Half_Reverse = half reverse (NBD–TMD); NBD_only = NBD-only soluble proteins.*

Table S7. MusiteDeep PTM predictions cross-referenced against UniProt experimental annotations for human ABC transporters.

MusiteDeep predictions (score ≥ 0.5) for 47 human ABC transporters were cross-referenced against experimentally annotated PTM sites (ECO:0000269) from UniProt Swiss-Prot (positional tolerance ±2 residues). Experimental PTM coverage in UniProt is sparse for most subfamily members and concentrated in well-characterised transporters including CFTR, ABCG1, ABCG2, MRP1, and ABCB11; unverified sites reflect absence of published experimental evidence at the time of analysis rather than confirmed false positives. Columns: Gene, UniProt accession, Position, Residue, PTM_type, Experimentally_verified (TRUE if a matching UniProt annotation was recovered within ±2 residues), PubMed_IDs (verified sites only), Protein_name.

Gene	UniProt	Position	Residue	PTM type	Recovered
ABCF1	Q8NE71	109	S	Phosphoserine	Yes
ABCF1	Q8NE71	140	S	Phosphoserine	Yes
ABCG1	P45844	30	C	S-palmitoyl cysteine	Yes
ABCG1	P45844	154	C	S-palmitoyl cysteine	Yes
ABCG1	P45844	315	C	S-palmitoyl cysteine	Yes
ABCG1	P45844	394	C	S-palmitoyl cysteine	Yes
ABCG1	P45844	406	C	S-palmitoyl cysteine	Yes
CFTR	P13569	524	C	S-palmitoyl cysteine	Yes
CFTR	P13569	660	S	Phosphoserine	Yes

CFTR	P13569	670	S	Phosphoserine	**Yes**
CFTR	P13569	686	S	Phosphoserine	**Yes**
CFTR	P13569	688	K	Ubiquitination	**Yes**
CFTR	P13569	700	S	Phosphoserine	**Yes**
CFTR	P13569	712	S	Phosphoserine	**Yes**
CFTR	P13569	717	T	Phosphothreonine	**Yes**
CFTR	P13569	737	S	Phosphoserine	**Yes**
CFTR	P13569	753	S	Phosphoserine	**Yes**
CFTR	P13569	768	S	Phosphoserine	**Yes**
CFTR	P13569	790	S	Phosphoserine	**Yes**
CFTR	P13569	795	S	Phosphoserine	**Yes**
CFTR	P13569	813	S	Phosphoserine	**Yes**
CFTR	P13569	1395	C	S-palmitoyl cysteine	**Yes**
CFTR	P13569	1442	S	Phosphoserine	**Yes**
CFTR	P13569	1444	S	Phosphoserine	**Yes**
CFTR	P13569	1456	S	Phosphoserine	**Yes**

Note. *Two experimentally verified sites were not recovered by MusiteDeep at the ≥ 0.5 score threshold (2/27 = 7.4% false-negative rate). CFTR (P13569) dominates the verified set because it is the most extensively characterised human ABC transporter for regulatory phosphorylation. The validation set is restricted to modification types classifiable under the MusiteDeep prediction categories; disulfide bonds and unclassified modifications were excluded.*
